# Effects of H_2_:CO_2_ ratio and H_2_ supply fluctuation on methane content and microbial community composition during in-situ biological biogas upgrading

**DOI:** 10.1186/s13068-019-1443-6

**Published:** 2019-04-30

**Authors:** Radziah Wahid, Daniel Girma Mulat, John Christian Gaby, Svein Jarle Horn

**Affiliations:** Faculty of Chemistry, Biotechnology, and Food Science, Norwegian University of Life Sciences (NMBU), P.O. Box 5003, 1432 Ås, Norway

**Keywords:** In-situ, Glucose, Mesophilic, Hydrogenotrophic methanogenesis, Biomethane

## Abstract

**Background:**

Commercial biogas upgrading facilities are expensive and consume energy. Biological biogas upgrading may serve as a low-cost approach because it can be easily integrated with existing facilities at biogas plants. The microbial communities found in anaerobic digesters typically contain hydrogenotrophic methanogens, which can use hydrogen (H_2_) as a reducing agent for conversion of carbon dioxide (CO_2_) into methane (CH_4_). Thus, biological biogas upgrading through the exogenous addition of H_2_ into biogas digesters for the conversion of CO_2_ into CH_4_ can increase CH_4_ yield and lower CO_2_ emission.

**Results:**

The addition of 4 mol of H_2_ per mol of CO_2_ was optimal for batch biogas reactors and increased the CH_4_ content of the biogas from 67 to 94%. The CO_2_ content of the biogas was reduced from 33 to 3% and the average residual H_2_ content was 3%. At molar H_2_:CO_2_ ratios > 4:1, all CO_2_ was converted into CH_4_, but the pH increased above 8 due to depletion of CO_2_, which negatively influenced the process stability. Additionally, high residual H_2_ content in these reactors was unfavourable, causing volatile fatty acid accumulation and reduced CH_4_ yields. The reactor microbial communities shifted in composition over time, which corresponded to changes in the reactor variables. Numerous taxa responded to the H_2_ inputs, and in particular the hydrogenotrophic methanogen *Methanobacterium* increased in abundance with addition of H_2_. In addition, the apparent rapid response of hydrogenotrophic methanogens to intermittent H_2_ feeding indicates the suitability of biological methanation for variable H_2_ inputs, aligning well with fluctuations in renewable electricity production that may be used to produce H_2_.

**Conclusions:**

Our research demonstrates that the H_2_:CO_2_ ratio has a significant effect on reactor performance during in situ biological methanation. Consequently, the H_2_:CO_2_ molar ratio should be kept at 4:1 to avoid process instability. A shift toward hydrogenotrophic methanogenesis was indicated by an increase in the abundance of the obligate hydrogenotrophic methanogen *Methanobacterium*.

**Electronic supplementary material:**

The online version of this article (10.1186/s13068-019-1443-6) contains supplementary material, which is available to authorized users.

## Background

Anaerobic digestion of biomass typically produces biogas with 50 to 70% CH_4_ and 30 to 50% CO_2_ [1–3]. Biogas may be upgraded to contain more than 90% CH_4_, which has higher heating value and can be used as a vehicle fuel or injected into natural gas grids [2]. Recently, interest has developed in upgrading the biogas through biological reduction of CO_2_ into CH_4_ by addition of exogenous hydrogen (H_2_) [4–9]. The microbial communities found in anaerobic digesters contain hydrogenotrophic methanogens, which use H_2_ as a reducing agent for the conversion of CO_2_ into CH_4_. Addition of H_2_ to such digesters has been shown to increase overall CH_4_ yield and to result in CH_4_ content above 90% [2, 10, 11]. The CO_2_ losses to the environment with commercial upgrading technology (scrubbing, pressure swing adsorption and membrane separation) can be minimized through biological biogas upgrading (BBU) by converting the CO_2_ into CH_4_ [12]. BBU may serve as a low capital cost upgrading technology because it can be easily integrated with existing biogas plants [4]. Moreover, this technology can be applied under mild operating conditions [13], without the need for high pressure or temperature. However, the present expense of H_2_ production is a limitation that must be overcome for large-scale application to become feasible.

An approach to reduce the H_2_ production cost is to utilize excess electricity produced from renewable sources such as wind and solar power [5] and convert it into H_2_ by water electrolysis. Water electrolysis is the only environmentally friendly technology for large-scale production of H_2_ [10]. However, the low density of H_2_ necessitates infrastructure to support its high storage volume. While the direct utilization of H_2_ as transport fuel remains under development [5, 10], the infrastructure for large-scale storage and utilization of CH_4_ (or natural gas) is already in place [14]. Thus, BBU may become a key technology for the storage of excess renewable electricity in the form of CH_4_.

Biological biogas upgrading can be implemented either in situ, where H_2_ is directly injected into anaerobic digesters or ex situ, where upgrading occurs in a separate reactor containing enriched cultures of hydrogenotrophic methanogens [8, 15–17]. Both approaches have allowed increases in CH_4_ content up to 90% and higher [2, 11]. In situ BBU is an attractive, low-cost option since it does not require investment for a second reactor and can be easily integrated with the existing anaerobic reactor at biogas plants [4, 18]. However, volumetric CH_4_ production rates are usually higher for ex situ BBU [8, 19, 20]. Maintaining stable reactor performance during in situ biogas upgrading is challenging due to several factors such as depletion in the buffering capacity of CO_2_ and high H_2_ partial pressure, which may lead to an increase in pH and volatile fatty acid (VFA) accumulation [21, 22]. Rigorous bioprocess development is required to improve in situ BBU.

The rate-limiting step in BBU is the low solubility of H_2_ in the aqueous reactor environment, which hinders H_2_ uptake by hydrogenotrophic methanogens [8, 23]. One approach to improve H_2_ availability in aqueous media is to increase the residence time of injected H_2_ by using batch reactors [5, 19, 20, 23–26]. In this approach, H_2_ is injected directly into the headspace of the reactor, which creates a concentration gradient from the headspace into the liquid phase, thus increasing H_2_ availability and thereby facilitating increased uptake of H_2_ by hydrogenotrophic methanogens [5, 23]. Szuhaj et al. [23] demonstrated a favourable outcome on BBU using fed-batch fermentation system where they achieved an increase in CH_4_ content from 18 to 80% with 99% H_2_ utilization. Mulat et al. [5] reported an increase of CH_4_ content up to 89% due to H_2_ addition and a decrease in CO_2_ content from 60 to 11%. Both experiments were performed with a gas retention time of 24 h. Voelklein et al. [17] examined the influence of gas retention time on ex situ BBU using a 9.5-L reactor. It was observed that the gas conversion peaked at the highest gas retention time (24 h). Under these conditions, the CH_4_ concentration was upgraded to 96% with 2.9% and 1.6% of CO_2_ and H_2_ remaining in the gas mixture, respectively. Biogas upgrading of this sort may be implemented easily at existing biogas plant facilities because of its simplicity.

While BBU clearly has demonstrated potential to upgrade biogas, knowledge about effects on the microbial community is still limited [27]. Such knowledge is important because the changes in the microbial community are one of the bottlenecks for CH_4_ enrichment in in situ BBU [27, 28]. Hence, the aims of the present study were to evaluate the influence of in situ BBU at mesophilic conditions (37 °C) on gas yield, gas composition, pH, VFA, total ammonium nitrogen (TAN), and total chemical oxygen demand (TCOD). During the experiment, the H_2_ retention time was kept at 24 h and the H_2_:CO_2_ ratio was gradually increased until maximum H_2_ conversion was observed. The in situ BBU was examined for different phases; start-up, initial H_2_ addition and inhibition, experimental and stable phases. Moreover, the effects on the microbial community throughout the different phases were investigated. The influence of variable H_2_ inputs on CH_4_ production was also evaluated in order to simulate a scenario where fluctuating excess electricity produced from wind and solar are used to generate H_2_.

## Results and discussion

### Reactor performance

**Fig. 1:**
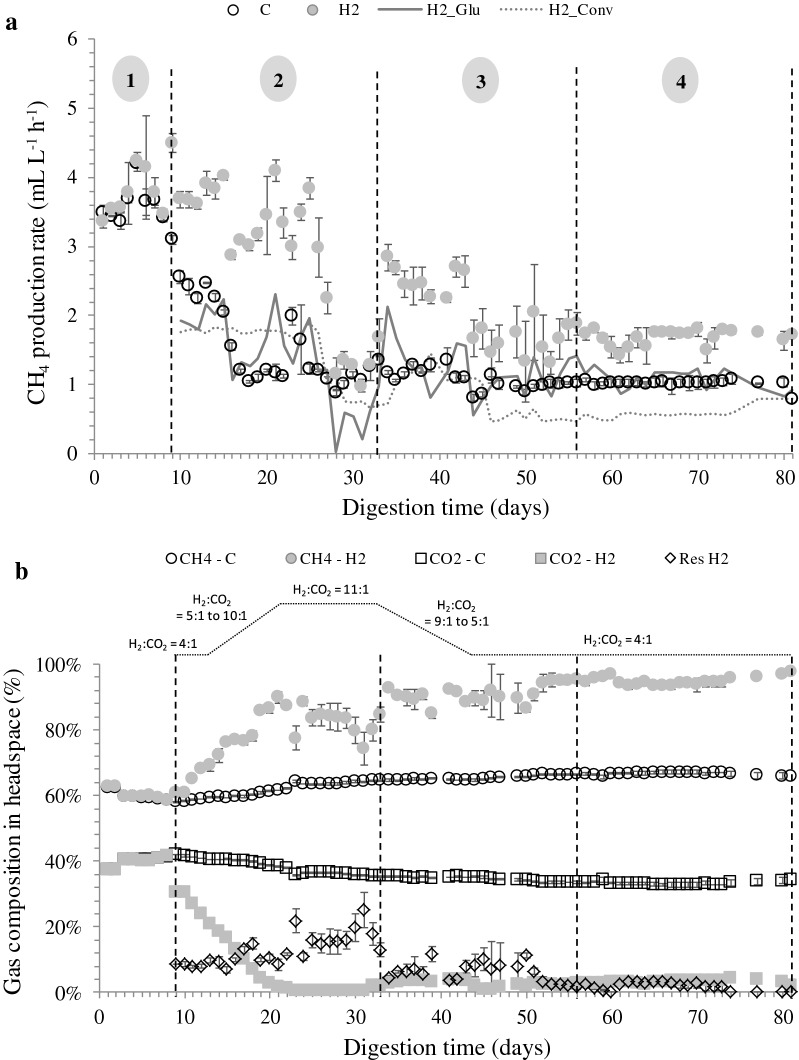
The average (**a**) CH_4_ production rate and (**b**) gas compositions of control and H_2_-supplemented reactors. 1—start-up phase, 2—initial H_2_ and inhibition phase, 3—experimental phase and 4—stable phase. C: control reactor; H_2_: H_2_-supplemented reactor; H2_Glu: CH_4_ production rate from glucose digestion; H2_Conv: CH_4_ production rate from H_2_ conversion; Res H_2_: residual H_2_

The performance of all reactors was analysed based on four phases: phase 1—start-up (day 1 to 9), phase 2—initial H_2_ addition and inhibition phase (day 10 to 33), phase 3—experimental phase (day 34 to 56) and phase 4—stable phase (day 57 to 81) (Fig. 1). We also define day 81 as the final timepoint in the context of the microbial community analyses, beyond which the composition began to diverge from the stable phase composition. At start-up, OLR for both reactors was changed twice due to overloading of glucose fed to the reactors, causing a drop in CH_4_ content up to 58% (day 1–9). Reduction of CH_4_ content due to glucose overload has been reported previously [29]. The calculated anaerobic biodegradability during this period was less than 40% in all reactors (data not shown). Later, at an OLR rate of 0.05 g_COD_ L^−1^ day^−1^, anaerobic biodegradability was improved to nearly 100%. Moreover, at this OLR, the CH_4_ content in the control reactors was increased to 67%.

### Start-up phase

**Fig. 2:**
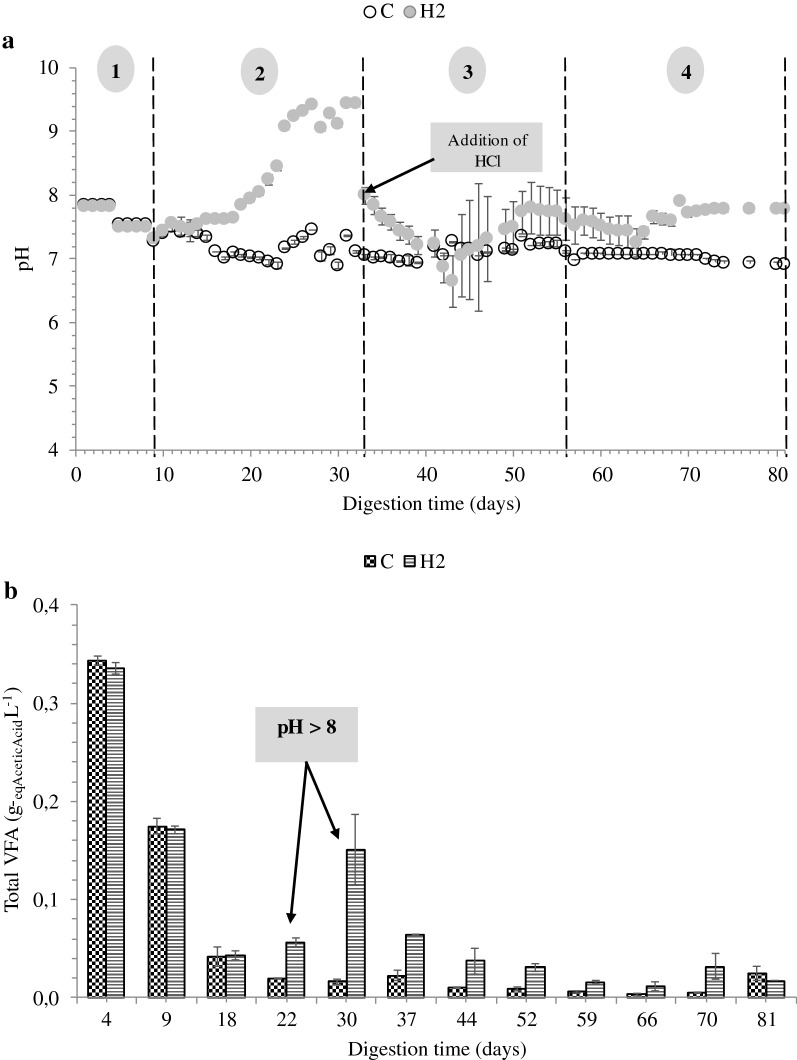
**a** pH and **b** total volatile fatty acid concentrations of the control and H_2_-supplemented reactors. C: control reactor; H_2_: H_2_-supplemented reactor

In this phase, both sets of reactors were running at similar operating conditions without addition of H_2_ until at day 9, when H_2_ was supplied to three of the reactors. The CH_4_ production rate, gas compositions, pH and VFA concentrations of both reactors were comparable during this phase (Figs. 1 and 2). The average CH_4_ production rates from control and H_2_-supplemented reactors were 3.61 ± 0.27 and 3.73 ± 0.32 mL L^−1^ h^−1^ (Fig. 1a), respectively. Methane content ranged from 58 to 63% while CO_2_ content ranged from 37 to 42% (Fig. 1b). The initial pH for both reactors was adjusted to 7.80, and after 4 days of anaerobic digestion, the pH dropped to 7.50 (days 5 to 8) and further reduced to 7.30 at day 9 (Fig. 2a). The drop in pH may have been due to glucose overloading at the OLR of 0.23 g_COD_ L^−1^ day^−1^, which corresponded to a drop in CH_4_ content from 63 to 58% (Fig. 2b).

### Initial H_2_ addition and inhibition phase

H_2_ uptake by methanogens was rapid as 93% of H_2_ was consumed within 24 h upon the first H_2_ injection (data not shown). The fast H_2_ consumption is in accordance with observations by Agneessens et al. [4] and Kern et al. [30] who noted that this occurred without any prior pre-enrichment of hydrogenotrophic methanogens. During the second phase, the CH_4_ production rate of the H_2_-supplemented reactors ranged from 2.87 to 4.10 mL L^−1^ h^−1^ (Fig. 1a). In this period, the H_2_:CO_2_ ratio was gradually increased up to an 11:1 ratio in an attempt to achieve full conversion of CO_2_ into CH_4_ (Fig. 1b). However, depletion of CO_2_ adversely affected reactor performance as the pH in H_2_-supplemented reactors increased to more than 8.5 with a maximum recorded pH of 9.4 (Fig. 2a). The optimal pH for mesophilic biogas reactors is reported in the range of 6.7 to 7.4, while methanogens have optimum growth between pH 7.0 to 8.0 [31]. An excessively alkaline pH may limit the methanogenic activity, which may lead to process inhibition and decreased CH_4_ production [31]. This fact was corroborated by the reduction in CH_4_ production rate observed from day 26 to 31 (Fig. 1a). The CH_4_ production rate from glucose (H2_Glu) dropped in this period sharply from 1.9 to 0 mL L^−1^ h^−1^ indicating severe inhibition of the anaerobic digestion process. Similar observations have been reported previously [32, 33] where reactors supplied with H_2_:CO_2_ ratios above 4:1 led to an increase in pH as CO_2_ was depleted. Furthermore, Agneessens et al. [4] observed a strong increase in pH when H_2_ was supplied at a H_2_:CO_2_ ratio of 10:1.

Besides CO_2_ depletion, high H_2_ partial pressure (11 to 25% residual H_2_) in the reactors due to H_2_ overloading may also explain the reduction in CH_4_ yield [34]. The H_2_ partial pressure during this period reached a maximum of 25 mbar. Hydrogen partial pressures exceeding 10^−3^ mbar may disturb propionate conversion to acetate, leading to accumulation of intermediate products and in extreme cases to complete process failure [17, 21, 35]. Additionally, Ahring et al. [36] reported that high H_2_ partial pressure (> 2.5 × 10^−3^ mbar) inhibits *Methanosarcina* and restricts acetate consumption. In our study, total VFA concentrations in the H_2_-supplemented reactors were increased from 0.04 to 0.15 g L^−1^ (Fig. 2b). The acetic and propionic acid increased from 0.004 to 0.096 g L^−1^, and from 0.028 to 0.048 g L^−1^, respectively. Similar findings were reported by Mulat et al. [5], where VFA levels were higher when H_2_ was added in excess into batch reactors, demonstrating the negative influence of excess H_2_ supply on the degradation of VFAs.

### Experimental phase

As the pH of the H_2_-supplemented reactors rose, the reactors were fed with an acidified glucose solution from day 33 until 43, causing a pH reduction from 9.4 to 6.6. As a result, the VFA concentration decreased by 40% while the CH_4_ production rate increased beginning on day 34. The CH_4_ concentration also rose from 85 to 93%. The H_2_:CO_2_ ratio was adjusted to 4:1 after 45 days and kept constant until the end of the digestion period. Our results agree with previous research by Wang et al. [33]. These authors observed that BBU was severely inhibited when the gas injection rate was increased from 1300 (H_2_:CO_2_ ratio ~ 4:1) to 2882 mL day^−1^ (H_2_:CO_2_ ratio ~ 8:1). Similarly, rapid recovery of BBU was observed after addition of HCl into the reactor, indicating the robustness of BBU and the importance of pH control for achieving high CH_4_ content and efficient H_2_ conversion.

### Stable phase

The stable phase of the H_2_-supplemented reactors began at day 57 and lasted until the end of the experiment. The average CH_4_ production rate and CH_4_ content from the reactor supplied with H_2_ were significantly higher than for the control reactor (Fig. 1a and b). It was noted that the amount of CH_4_ from glucose degradation was comparable (Fig. 1a). The average CH_4_ production rate in the H_2_-supplemented reactor was 1.68 ± 0.11 mL L^−1^ h^−1^ (0.59 ± 0.09 mL L^−1^ h^−1^ calculated from H_2_ conversion) while it was 1.03 ± 0.02 mL L^−1^ h^−1^ in the control reactor (Table 1). Approximately 54% of the additional CH_4_ was calculated to result from H_2_ and CO_2_ conversion. The CH_4_ concentration in the H_2_-supplemented reactors increased from 66.7 to 94.5%, approximately 42% higher than the control. At the same time, CO_2_ levels decreased from 33.3 to 3.1% due to H_2_ addition, and the average residual H_2_ concentration was around 2.5%. Nearly 98% of H_2_ added into the reactors was utilized for conversion of CO_2_ into CH_4_. During this phase, the pH of both reactors decreased below 8, and the VFA level was gradually reduced towards the end of the experiment. The average pH and VFA concentration of the control reactors were 7.07 ± 0.11 and 35.4 ± 20.40 mg L^−1^, respectively, while the corresponding values for the H_2_-supplemented reactors were 7.64 ± 0.15 and 37.5 ± 10.40 mg L^−1^.

**Table 1:** Process performance variables for control and H_2_-supplemented reactors during the stable phase (mean ± S.D)

	Control	H_2_-supplemented
Biogas compositions (%)		
CH_4_	66.70 ± 0.36	94.47 ± 1.21
CO_2_	33.30 ± 0.36	3.09 ± 0.64
H_2_	–	2.45 ± 0.83
CH_4_ production rate (mL L^−1^ h^−1^)	1.03 ± 0.02	1.68 ± 0.11
CH_4_ from H_2_ consumed (mL L^−1^ h^−1^)	–	0.59±0.09
H_2_ added to reactor (mL L^−1^ h^−1^)	–	2.37 ± 0.39
H_2_ consumed (mL L^−1^ h^−1^)	–	2.32 ± 0.38
ɳH_2_ (%)	–	98
Total VFA (mg L^−1^)	35.4 ± 20.40	37.5 ± 10.40
pH	7.07 ± 0.11	7.64 ± 0.15

Based on the data obtained during stable phase, the mass balance of CH_4_ production from glucose with and without H_2_ addition was calculated following the procedure of Mulat et al. [5]. The mass balance calculation considered (1) the measured CH_4_ yield from the experiment, (2) the CH_4_ yield expected from the stoichiometric H_2_ consumption and (3) the potential CH_4_ yield from unconsumed VFAs in the reactors. The average CH_4_ yields per day were 9.55 mL in the control reactor and 15.67 mL in the H_2_-supplemented reactor (Table 2). It was estimated that addition of 21.75 mL of H_2_ into the reactor yielded 5.44 mL of CH_4_ based on the 4:1 H_2_:CO_2_ molar ratio. Potential extra CH_4_ yields estimated from unconverted VFAs in the control and the H_2_-supplemented reactors were 1.42 and 1.78 mL, respectively. Theoretical total CH_4_ yield for the H_2_-supplemented reactor, calculated from CH_4_ produced from the control reactor + CH_4_ estimated from unconsumed VFA + CH_4_ estimated due to H_2_ addition, was 16.41 mL, while the observed CH_4_ yield was 17.45 mL. Our calculation showed 100% recovery of the added H_2_ and glucose substrate in the form of CH_4_ produced and residual VFA.

**Table 2:** Mass balance of CH_4_ production, with and without H_2_ addition during the stable phase

Reactor ID	Amount of CH_4_ measured from experiment (mL)	Total H_2_ consumed (mL)	CH_4_ produced due to H_2_ addition (mL)^a^	CH_4_ estimated from residual VFA (mL)	Total CH_4_ produced (CH_4_ and residual VFA) (mL)	Theoretical CH_4_ (mL)^b^	Recovery (%)^c^
Control	9.55	0	0	1.42	10.97	–	–
H_2_-supplemented	15.67	21.75	5.44	1.78	17.45	16.41	106

^a^CH_4_ from H_2_ consumed based on the Sabatier equation. (4 mol of H_2_ are required to convert 1 mol of CO_2_ into CH_4_)

^b^Total CH_4_ production from the control reactor plus the CH_4_ produced due to H_2_ addition

^c^Recovery = (Theoretical CH_4_/Total CH_4_ produced from the H_2_-supplemented reactor) × 100

### Pulsed H_2_ feeding

To investigate the flexibility of the BBU system towards variable H_2_ input (fluctuating supply of H_2_), a test was conducted for 12 consecutive days (days 82 to 93) during stable phase using the three H_2_-supplemented reactors. During this test, the reactors were fed with glucose solution once daily at an OLR of 0.05 g_COD_ L^−1^ day^−1^, and H_2_ was injected into the reactor with the H_2_:CO_2_ ratio of 4:1. From day 82 to 87, addition of H_2_ into the reactors was performed every 24 h, and starting at day 88, H_2_ addition was ceased for 3 consecutive days. H_2_ supply was resumed from day 91 until day 93, with conditions similar to those prior to the cessation of H_2_ supply.

**Fig. 3:**
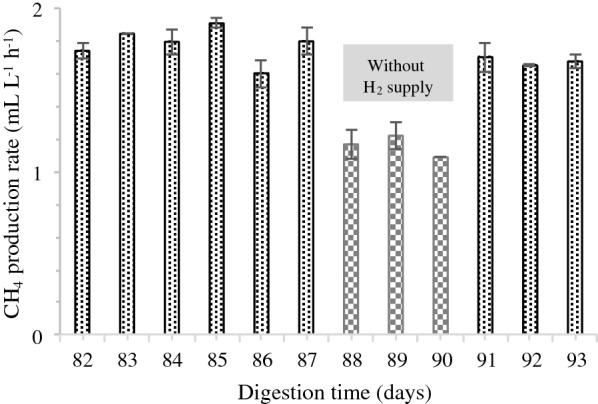
Methane production rate during continuous and intermittent H_2_ addition

The average CH_4_ production rate from the reactors fluctuated with the H_2_ supply (Fig. 3). When H_2_ supply ceased, the CH_4_ production rate dropped from 1.80 ± 0.10 to 1.20 ± 0.10 mL L^−1^ h^−1^ and then increased again to 1.70 ± 0.04 mL L^−1^ h^−1^ once H_2_ addition resumed on day 91. The average CH_4_ production rate under H_2_ supply in this fluctuation experiment was comparable with that observed during the normal supply of H_2_ at stable phase (Fig. 1a). In addition, the average CH_4_ production rate when H_2_ was not supplied was similar to the production rate of the control reactor. These observations demonstrate that the BBU system responds favourably to fluctuating H_2_ supply by not compromising CH_4_ yield and content. Moreover, the results indicate rapid response of hydrogenotrophic methanogens to conversion of CO_2_ and H_2_ into CH_4_. Agneessens et al. [4] reported a similar observation where initial H_2_ uptake rates increased during consecutive pulse injections of H_2_ into an anaerobic reactor.

Even though favourable outcomes were demonstrated in our study, one should keep in mind that the implementation of BBU at larger scales is not economical in the short term due to high investment costs [12]. Currently, BBU seems to be more expensive than the cost of CO_2_ removal per kWh of biomethane produced [37]. The total cost of BBU is greatly influenced by the H_2_ production costs, which are estimated to be around 0.09 €/kWh [37]. However, in the long term, it is expected that there will be increased incentives for upgrading biogas through the conversion of H_2_ and CO_2_. The decline in renewable electricity production costs (solar and wind) is predicted to continue, which will contribute to decreased H_2_ costs [37]. The high efficiency of biomethanation, the low feedstock prices and the potential for CH_4_ prices to increase are the other factors that may lead to BBU becoming a more economical process [12].

### Microbial community

**Fig. 4:**
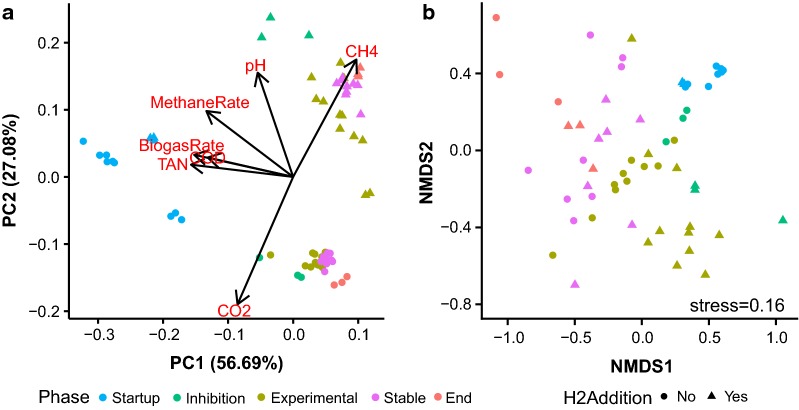
**a** PCA analysis of select process parameters whose identities are indicated by the red text and **b** NMDS ordination of microbial community composition in each sample based on Bray–Curtis distances calculated from ASV abundances. The variation explained by the first two principal components is indicated in the axis labels (**a**) and the stress for the NMDS ordination is indicated by the text in (**b**). Samples are coloured by phase of reactor operation while samples from the control reactor are distinguished from those from the hydrogen-supplemented reactor by symbol shape as indicated in the legend at the bottom of the figure. Samples with less than 1000 sequences were omitted from the NMDS ordination (**b**)

We applied the multivariate statistical procedure principal component analysis (PCA) to compare reactor timepoints according to their unique set of process parameter values (Fig. 4a), and we additionally analysed the microbial community composition for each reactor timepoint by performing non-metric multidimensional scaling (NMDS) ordination on the Bray–Curtis distances (Fig. 4b), a metric which considers both the species present in a sample as well as their abundance. The PCA analysis allows assessment of the similarity in reactor environment for the different reactor timepoints (Fig. 4a), whereas the NMDS analysis evaluates similarity in the microbial community composition across reactor timepoints (Fig. 4b), and comparing the process parameter PCA plot with the microbial community, NMDS plot allows for inference of the process parameters that correspond to changes in microbial community composition. The characteristics of each reactor environment changed over the time course of the experiment (Fig. 4a), as did the microbial community composition (Fig. 4b) in the reactors. The reactor timepoints were grouped into (1) start-up phase (days 4 and 9), (2) inhibition phase (day 30), (3) experimental phase (days 37, 44, 45 and 52), (4) stable phase (days 59, 66 and 70) and (5) end phase (day 81). The timepoints cluster together by phase and form a continuum from start-up phase to end phase, which indicates that the reactor conditions and the microbial community gradually changed over time. The two main principle components (PC) explain 84% of the variation in the process parameter data, and the NMDS ordination has a stress of 0.16 which indicates that the plot provides an acceptable two-dimensional representation of the Bray–Curtis distances (Fig. 4b). The CO_2_ and CH_4_ concentrations in the reactor biogas clearly separate the reactors with H_2_ addition from the control reactors (Fig. 4a), but it is not as apparent in the microbial data (Fig. 4b). Also, the TAN, COD, and biogas production rate distinguish the start-up phase from the other phases. An increase in pH in the H_2_-supplemented reactors corresponds to the divergence of its microbial community (Fig. 4b). Hence, stabilization of pH (Fig. 2a) and VFA concentrations (Fig. 2b) after about day 40 resulted in another shift in the microbial community.

**Fig. 5:**
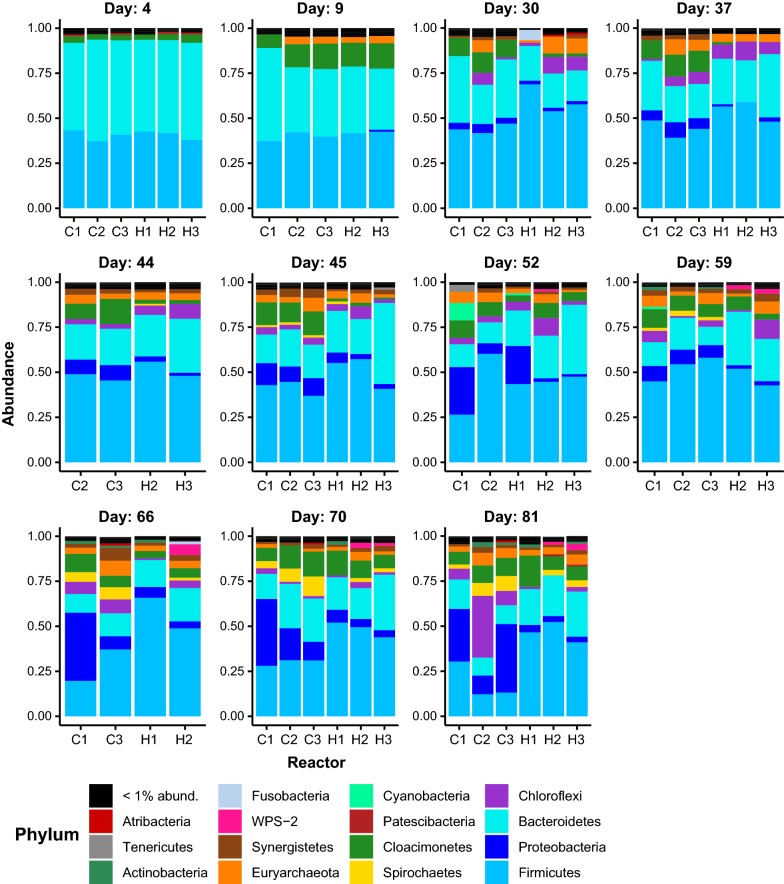
Stacked barplot of the relative abundance of phyla detected in each sample. Phyla are indicated by the colours displayed in the legend at the bottom of the figure. Reactor type and replicate are indicated in the horizontal axis labels in each facet (e.g. C1 is control reactor replicate 1, and H indicates the hydrogen-supplemented reactors). The text at the top of each facet indicates the sampling timepoint in days since reactor start-up. Samples with less than 1000 sequences were omitted from the figure

Both the distribution of bacterial phyla (Fig. 5) and ASVs (Amplicon Sequence Variants; Additional file 1: Figure S1) changed during the experiment. Initially, *Bacteroidetes* dominated both reactors, followed by *Firmicutes* and *Cloacimonetes*. The abundance of *Bacteroidetes* and *Firmicutes* accounted for almost 90% of the bacterial sequence reads. *Bacteroidetes* and *Firmicutes* are typically the most abundant bacterial phyla in biogas reactors [28, 38]. During digester operation, the abundance of other phyla including *Chloroflexi, Spirochaetes, Proteobacteria, Euryarchaeota, Actinobacteria* and *Synergistetes* increased in both reactors. The WPS-2 phylum was also observed in the H_2_-supplemented reactors. In accordance with previous studies, *Firmicutes* dominate H_2_-supplemented reactors and account for approximately 40% of the microbial community [2, 39, 40]. *Firmicutes* are involved in various metabolic processes for the degradation of carbohydrates and fatty acids, including the Wood–Ljungdahl pathway (homoacetogenesis) and syntrophic acetate oxidation [39, 41], which may explain the dominance of this phylum in the H_2_-supplemented reactors. Compared to the H_2_-supplemented reactors, the control reactors had lower abundance of *Firmicutes* and *Bacteroidetes*, and a higher abundance of *Proteobacteria* and *Chloroflexi* was observed in them at day 81. Both *Proteobacteria* and *Chloroflexi* are known as important taxa that consume glucose in digesters [42, 43].

During day 23 to 34 the pH was high in the H_2_-supplemented reactors relative to the controls (Fig. 2b), and from day 32 to 43 acid was added to the H_2_-supplemented reactors to equilibrate the pH to that of the controls. Some bacteria in the H_2_-supplemented reactors, such as the unidentified species of *Rikenellaceae* DMER64, corresponding to ASV2 (Additional file 1: ASV Catalogue S1), clearly responded during this period of pH disturbance, but quickly returned to the abundances found in the control reactor after the pH again returned to below 8. *Rikenellaceae* is a fermentative anaerobic microorganism which is involved in VFA (acetate, succinate, propionate), NH_3_, CO_2_ and H_2_ production [28]. It has previously been detected in mesophilic biogas reactors [39, 44] and has been shown to be negatively affected by H_2_ addition [44]. Our results confirmed the adverse effect of H_2_ on *Rikenellaceae*, as a sharp decline in its relative abundance was observed at high H_2_:CO_2_ ratios. Interestingly, when the H_2_:CO_2_ ratio was adjusted to 4:1, *Rikenellaceae* responded positively and its abundance continuously increased until day 81. A pH below 8 is favourable for *Rikenellaceae*, and the lower partial pressure of H_2_ at the 4:1 H_2_:CO_2_ ratio is also beneficial for the H_2_ producing pathways of *Rikenellaceae*.

**Fig. 6:**
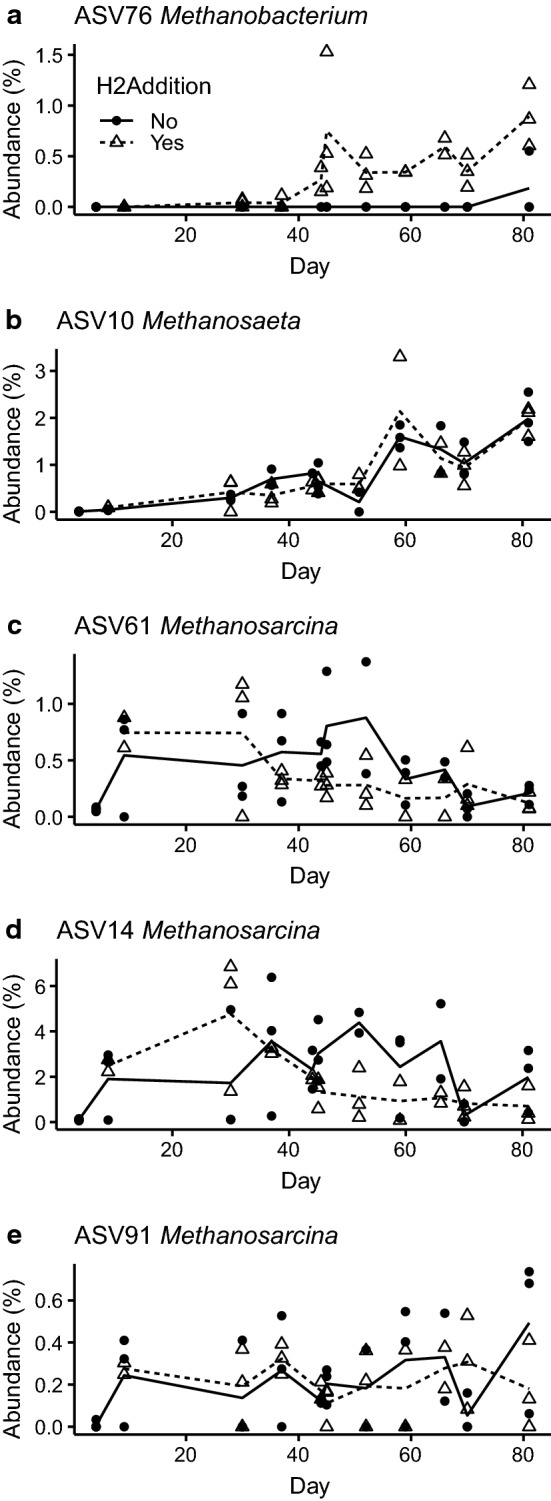
Percent abundance of 5 methanogen ASVs in the control and hydrogen-supplemented reactors. The hydrogenotrophic *Methanobacterium* (**a**), acetoclastic *Methanosaeta* (**b**) and 3 ASVs of *Methanosarcina* (**c**–**e**), which may carry out both forms of methanogenesis, were detected among the top 100 most abundant ASVs. Datapoints in each figure indicate the abundance of the ASV in a reactor replicate sample at the time in days since reactor start-up. The solid line indicates the mean of control reactor abundances and the dashed line indicates that for the hydrogen-supplemented reactors, while symbol shape and fill indicate whether the datapoint corresponds to a reactor without H_2_ addition (control) or with (H_2_ supplemented), as indicated in the legend in the plot (**a**). Samples with less than 1000 sequences were omitted from the figure

In contrast to this transient divergence, the abundance of some microorganisms remained divergent after the spike in pH had subsided, supporting an effect of H_2_ addition on those particular taxa. For instance, the hydrogenotrophic methanogen *Methanobacterium* sp. (ASV76, Fig. 6a) followed this pattern. *Methanobacterium* sp. is a dominant methanogen for biogas production via syntrophic acetate oxidation and hydrogenotrophic methanogenesis [28] and has been observed in sugar-processing wastewater plants [45]. *Methanobacterium* sp. was detected in the H_2_-supplemented reactors by day 30, increased in abundance thereafter and continued to be detected in the H_2_-supplemented reactors, but was not present in the control reactors throughout the course of the experiment (Fig. 6a). The enrichment of *Methanobacterium* sp. in the H_2_ fed reactors has also been observed by Mulat et al. [5] and Rachbauer et al. [28], demonstrating a general shift towards hydrogenotrophic methanogens. The shift from acetoclastic to hydrogenotrophic methanogenesis was expected, agrees well with previous observations and shows that enrichment of hydrogenotrophic methanogens is possible under these conditions.

*Methanosaeta* sp. (ASV10, Fig. 6b) is an acetoclastic methanogen whose abundance was consistent in both the control and H_2_-supplemented reactors. Its abundance appeared to increase over the duration of the experiment in both reactor types. *Methanosaeta* sp. are obligate acetoclastic methanogens and are mainly observed in mesophilic anaerobic reactors [46]. *Methanosaeta* sp. has a relatively slow growth rate but possesses a high affinity for VFAs, and it may thus dominate anaerobic reactors with low acetate concentrations [47]. This is in agreement with the present study, as *Methanosaeta* sp. was enriched when the VFA concentrations were reduced over time. This finding suggests that *Methanosaeta* sp. might be the main producer of CH_4_ from acetoclastic methanogenesis in the latter period of the AD, although definitive support of this supposition would require transcriptomic, proteomic, or stable isotope probing data.

*Methanosarcina* sp. was the only other detected methanogen capable of hydrogenotrophic methanogenesis present in the reactors, although it can also carry out acetoclastic methanogenesis [48]. Differences between the control and H_2_-supplemented reactors for ASV61 and ASV14 affiliated to this genus were transient and occurred around days 45 and 50 (Fig. 6c, d). *Methanosarcina* sp. was the dominant methanogen in both reactors at the start of the experiments. However, it is likely that the abundance of *Methanosarcina* sp. was negatively affected by the gradual reduction of the VFA and TAN concentrations. In the end phase, the abundance of *Methanosarcina* sp. (ASV14 and ASV61) was below 2% in both reactors. As mentioned previously, acetate utilization seemed to shift from *Methanosarcina* sp. (ASV14 and ASV61) in the early phase to *Methanosaeta* sp. at the later stage of the experiment. This finding agrees with previous studies [42, 49, 50], thereby demonstrating high abundance of *Methanosarcina* sp. at elevated VFA concentrations, while *Methanosaeta* sp. dominated under low VFA concentrations.

It is likely that *Methanosarcina* sp. (ASV14 and ASV61) was inhibited by the high H_2_ partial pressure and high pH in the H_2_ fed reactors. This observation was corroborated by the reduced CH_4_ production in this period along with the VFA accumulation. Previously, it has been reported that *Methanosarcina* sp. was negatively affected by high H_2_ partial pressure [36], and the optimum pH for *Methanosarcina* sp. lies between 5 and 8 [51]. It is known [4] that an increase in pH favours the conversion of H_2_ to acetate by homoacetogens and reduces the activity of acetoclastic methanogens. Our study confirmed this as the abundance of *Methanosarcina* sp. decreased after day 30. The abundance of ASV91, also affiliated to this genus, did not significantly diverge between the control and H_2_-supplemented reactors (Fig. 6e).

ASV42, ASV64 and ASV67 (Additional file 2: ASV Catalogue) emerged after day 30 in the H_2_-supplemented reactors and correspond to species of *Sporomusa*, and a strain of this species has been characterized as a homoacetogen [52]. Homoacetogens are H_2_ consumers and are able to grow at the expense of H_2_ and CO_2_ as sole energy source [53]. They do not compete well with methanogens because of their less favourable thermodynamic characteristics. The affinity of methanogens for H_2_ was shown to be 10–100 times higher than that for homoacetogens [53]. However, if methanogens were inhibited, then the accumulation of H_2_ might be favourable for homoacetogenesis. Chen et al. [54] observed significant acetate accumulation and low CH_4_ yield when H_2_ and CO_2_ were supplied as substrate to a municipal solid waste landfill, indicating that homoacetogens outcompeted hydrogenotrophic methanogens in the landfill samples. The finding was comparable with our study as *Sporomusa* was only detected starting at day 30 (inhibition phase). Acetate accumulation and low CH_4_ production were observed during this period. It has been reported previously that the addition of H_2_ into anaerobic reactors results in an increase in homoacetogen abundance [44]. Acetate formed by this process may support acetotrophic methanogenesis, although this is not supported by our observations given that the acetoclastic methanogens did not increase in abundance in the H_2_-supplemented reactors relative to the control reactors (Fig. 6b–e). The acetate produced by *Sporomusa*, however, may enter into catabolic pathways and support growth and the corresponding increase in abundance observed in the *Sporomusa* ASVs in the present study.

ASV23, ASV32 and ASV52 (Additional file 2: ASV Catalogue) correspond to a species designated W5 within the *Cloacimonadaceae* family. Their abundance increased in the control reactors above that of the H_2_-supplemented reactors, a difference that persists well through the stable phase until day 70 (Additional file 2: ASV Catalogue) and reached 1 to 3% maximum abundance. The related ASV5 *Candidatus* Cloacimonas reached up to 15% abundance but only exhibited divergence between the H_2_ and control reactors during the period of pH increase whereby it decreased in the H_2_-supplemented reactors from day 30 to 45. *Cloacimonadaceae W5* and *Candidatus* Cloacimonas are known to be anaerobic mesophilic acetogens [55]. High abundance of *Cloacimonadaceae* W5 and *Candidatus* Cloacimonas in biogas reactors has been observed previously [55, 56].

There are several ASVs with noteworthy abundance changes (Additional file 2: ASV Catalogue). ASV25, whose abundance increased in the control reactors from day 30 onward, corresponds to *Smithella*, species of which are known propionate oxidizing bacteria [57]. *Smithella* remained at low abundance in the H_2_-supplemented reactors during the 70 days of reactor operation. *Smithella* was the dominant species in a mesophilic reactor and its abundance was reduced to 40% due to H_2_ addition [33]. It was reported that propionate oxidizing bacteria are highly sensitive and can be easily inhibited by increases of H_2_ in the system [58].

ASV31 is an uncharacterized order of *Clostridia* named DTU014 and exhibited a persistent increase due to H_2_ addition. *Clostridia* DTU014 has been observed in biogas reactors and plays an important role in oligosaccharide and monosaccharide utilization [39]. ASV 44 (*Desulfovibrio*) and ASV66 (*Desulfomicrobium*) emerged and persisted in the control reactor. Both ASVs are sulphate-reducing bacteria that utilize H_2_ or organic matter as an electron donor and sulphate as an electron acceptor. It has been reported that under standard conditions, sulphate-reducing bacteria outcompete methanogens and homoacetogens as H_2_ consumers [59]. However, H_2_ was previously observed to be mainly consumed by a sulphate reducer under H_2_ limiting conditions in the presence of sufficient sulphate [60]. Our results support this prior observation as *Desulfomicrobium* and *Desulfovibrio* abundances were suppressed at high H_2_ partial pressure, indicating that H_2_ was mainly utilized by methanogens or homoacetogens during BBU. ASV94 is an uncharacterized organism in the *Selenomonadales* order of the *Firmicutes*, and it emerged first in the H_2_-supplemented reactors after day 50 and reached a maximum of 2% abundance.

## Conclusions

This study demonstrated the feasibility of biogas upgrading through biological means in a batch reactor and showed that addition of H_2_ into a batch reactor increased CH_4_ content from 67 to 94%. H_2_:CO_2_ ratios above 4:1 in the system fully utilized CO_2_, yet led to process imbalance due to bicarbonate consumption and high H_2_ partial pressure. This clearly indicated that the H_2_:CO_2_ ratio is an important parameter which should be kept at 4:1 to ensure stable upgrading with pH < 8 and low VFA concentrations. The resilience of biological biogas upgrading to fluctuating H_2_ inputs was demonstrated by immediate CO_2_ conversion into CH_4_ after pulsed H_2_ addition. In addition, the observed increase in abundance of *Methanobacterium*, a hydrogenotrophic methanogen, demonstrated that H_2_ addition selects for hydrogenotrophic methanogenesis.

## Methods

### Substrate and inoculum

A d-glucose (99.5%, Sigma-Aldrich) solution at 1% w/v concentration was used as a model substrate. Mesophilic inoculum was collected from a lab-scale 10 L digester at the biogas laboratory of the Norwegian University of Life Sciences (NMBU) in Ås, Norway. The mesophilic digester, running at 37 °C, was primarily digesting cattle manure collected from a cattle farm in Ås, Norway. The inoculum was filtered through a 1-mm sieve and degassed for 10 days at 35 °C to minimize the background biogas production. Substrate and inoculum characteristics are shown in Table 3.

**Table 3:** Inoculum characteristics (mean ± S.D, *N* = 3)

Parameters	Inoculum
Total chemical oxygen demand, tCOD (g L^−1^)	20.52 ± 1.01
Total solid, TS (% FM)	2.51 ± 0.12
Volatile solid, VS (% FM)	1.48 ± 0.10
pH	7.82
Total ammonium nitrogen (g L^−1^)	1.09 ± 0.04
Total volatile fatty acid (g L^−1^)	0.73 ± 0.04
Acetic acid (g L^−1^)	0.34
Propionic acid (g L^−1^)	0.23 ± 0.01
Butyric acid (g L^−1^)	0.02 ± 0.01
Iso-butyric acid (g L^−1^)	0.06 ± 0.01
Iso-valeric acid (g L^−1^)	0.07 ± 0.01

*FM* fresh matter

### In-situ batch fermentation

The batch experiments were carried out at 37 °C in 0.5 L glass bottles (diameter: 76 mm, height: 188 mm, Nordic pack, Sweden) operating at 21 days hydraulic retention time (HRT) for 81 days. Both control reactions (bottles without H_2_ addition) and reactions supplied with H_2_ were carried out in triplicate. Initially, the bottles (reactors) were loaded with 200 mL of manure-based inoculum. Water was added to the bottles to reach 388.5 mL working volume. After filling, the bottles were sealed with a butyl rubber stopper and aluminium crimp, and the headspace was flushed with pure N_2_ for 3 min. The experiment was conducted in an incubator shaker (Multitron Standard, Infors HT, Switzerland) with 100 rpm mixing speed. Every 24 h, 18.5 mL of 1% glucose solution was fed into the reactors after the same amount of effluent was discharged. For the first 8 days, all reactors were operated under the same conditions, but from day 9 onward, H_2_ was supplied to the three experimental reactors but not to the three control reactors. Before each H_2_ injection, the headspace pressure of the reactor was vented to atmospheric pressure. The ratio of H_2_ injected to daily produced CO_2_ ranged from 4:1 to 11:1 in the early phase (day 9 to 44) to achieve full CO_2_ conversion and later adjusted to 4:1 ratio (day 45 onwards). During the initial phase of reactor operation, the organic loading rate (OLR) was changed twice from 0.23 to 0.18 g_COD_ L^−1^ day^−1^ and from 0.18 to 0.05 g_COD_ L^−1^ day^−1^ by reducing the concentration of glucose. Nutrients were supplied to the reactors by replacing the liquid mixture with 10 mL diluted manure at days 31, 49, 55 and 65.

### Analytical methods and calculations

Prior to the H_2_ injections, which occurred every 24 h, gas volume and gas composition (CH_4_, CO_2_ and H_2_) were measured. The gas overpressure in the reactor was measured with a digital pressure meter (GMH 3161, Greisinger, Germany). Methane and CO_2_ contents were analysed using a gas chromatograph (GC) (3000 Micro GC, Agilent Technologies, USA) equipped with a thermal conductivity detector (TCD). The Micro GC utilizes two capillary columns for gas separation. The two types of columns are (1) Molecular Sieve 5A (MolSieve) and (2) PLOT Q. The temperature for the sample inlet was maintained at 60 °C for both columns. The injector and column temperatures for MolSieve were 90 and 70 °C while those for the PLOT Q were 50 and 45 °C, respectively. Helium was used as the carrier gas for the GC. GC calibration was performed before biogas measurement using standard mixture of CH_4_ and CO_2_ (AGA, Norway).

Methane production was expressed at standard conditions, (temperature = 0 °C, pressure = 1 atm) according to the recommendations made by Angelidaki and Sanders [61]. The volume of biogas was calculated using the ideal gas law as described previously [62]. The H_2_ content was determined by GC (HP 5890A, California, USA) equipped with a TCD and a capillary column CarboPLOT P7 from Varian. The injector and detector temperature was set at 150 °C while the oven temperature was set at 30 °C. Nitrogen was used as the carrier gas for the GC.

The H_2_ conversion efficiency (ɳH_2_) was calculated according to Eq. 1 [16]:

1 $$\upeta{\text{H}}_{ 2} \left( \% \right) \, = \, \left( {{\text{VH}}_{ 2} {\text{injected }}{-}{\text{VH}}_{ 2} {\text{residual}}} \right) / {\text{VH}}_{ 2} {\text{injected,}}$$

where VH_2_ injected is the volume of H_2_ (mL L^−1^ h^−1^) injected into the anaerobic reactor, and VH_2_ residual is the volume of H_2_ (mL L^−1^ h^−1^) left in the reactor after 24 h of H_2_ injection.

The pH of the liquid effluent was measured immediately after sampling to avoid CO_2_ loss from the liquid phase using a digital pH meter (Thermo Scientific Orion Dual Star, USA). Liquid effluent from the reactors was collected for TCOD, TAN and VFA analysis once per week. Total chemical oxygen demand was determined using a Merck Spectroquant^®^ COD cell test with 500–10,000 mg L^−1^ range and TAN was measured using the Merck Spectroquant^®^ photometric kit with 4.0–80.0 mg L^−1^ NH_4_-N range. The VFA analysis was performed using a previously described protocol [63] with some minor modifications as indicated below. Samples for VFA analysis were centrifuged at 14,000 rpm for 5 min. Approximately 10 µL of sulphuric acid (95% concentration) was added to the supernatant and then mixed before analysis by high-pressure liquid chromatography (HPLC) using a Dionex Ultimate 3000 system (Dionex, Sunnyvale, CA, USA) equipped with a UV detector and fitted with an Aminex^®^ HPX-87H column (300 × 7.8 mm and 9 µm particle size). The column was operated at 0.6 mL min^−1^ at 50 °C, and 1 µL of sample was injected. A gradient flow was applied using 4 mM H_2_SO_4_ as eluents. Based on the analysis, the concentration of individual VFAs such as acetic acid, propionic acid, butyric acid and valeric acid was determined by reference to dilution series of standards of known concentration.

### Microbial community analysis

#### DNA sampling and extraction

The liquid effluent (15 mL) from each reactor was collected weekly and stored at − 20 °C prior to DNA extraction. To obtain DNA for 16S amplicon sequencing, 1 mL of the liquid effluent was centrifuged and the pellet was used for DNA extraction. The template DNA was extracted using the PowerMag Soil DNA Isolation Kit (MO BIO Laboratories Inc., Carlsbad, CA, USA) on a KingFisher Flex DNA extraction robot (Thermo Fisher Scientific, Waltham, MA, USA). The DNA extraction was performed following the manufacturer protocol except that samples were subjected to bead beating at maximum intensity on a FastPrep-96 Homogenizer (MP Biomedicals LLC., Santa Ana, CA, USA) for 4 pulses of 30 s each and a 5-min interval between pulses. DNA template concentration was determined using a Qubit Fluorometer (Invitrogen/Thermo Fisher Scientific, Waltham, MA, USA), and these reactor digest DNA extracts were stored at − 20 °C until further use.

### 16S amplicon sequencing

The PCR amplification of the 16S rRNA gene for sequencing was performed using the primers Pro341F (5′- TCGTCGGCAGCGTCAGATGTGTATAAGAGACAGCCTACGGGNBGCASCAG -3′) and Pro805R (5′- GTCTCGTGGGCTCGGAGATGTGTATAAGAGACAGGACTACNVGGGTATCTAATCC -3′) that include the Illumina overhang adapter sequences on the 5′ end, which are specified in the Illumina protocol for 16S Metagenomic Sequencing Library Preparation (Part # 15044223 Rev. A), in addition to the underlined target-specific sequences from Takahashi et al. [64], which are designed to target both *Bacteria* and *Archaea*. The 25-µL PCR reactions contained 0.25 µM primers, 1× iProof High-Fidelity Master Mix (Biorad, Hercules, CA, USA), and 0.5 ng of reactor digestate DNA. The PCR thermal cycling consisted of a hot start step at 98 °C for 180 s followed by 25 cycles of 98 °C for 30 s, 55 °C for 30 s, 72 °C for 30 s and then a final 72 °C extension step for 300 s. The amplification quality was then confirmed by 1% agarose gel electrophoresis. A PCR clean-up was performed with Agencourt AMPure XP beads (Beckman Coulter Inc., Brea, CA, USA) according to the Illumina protocol for 16S Metagenomic Sequencing Library Preparation (Part # 15044223 Rev. A). A second PCR was carried out using Nextera XT Index Kit (Illumina Inc., San Diego, CA, USA), followed by PCR clean-up with Agencourt AMPure XP beads. The pool of purified index PCR products was quantified using a Qubit™ Fluorometer (Invitrogen, USA), and the final pool concentration was adjusted. The pool was then spiked with 15% PhiX control and an 8 pM denatured DNA library was prepared. The denatured library was sequenced on an Illumina MiSeq instrument using the Miseq Reagent kit V3.

### Sequencing analysis

The demultiplexed fastq files consisting of 16S amplicon reads were downloaded from the Illumina MiSeq and analysed with the DADA2 [65] and PhyloSeq [66] packages in R [67]. DADA2 parameters for the ‘filterAndTrim’ function included a truncation length of 300 for forward and 270 bases for reverse reads, a maximum of 4 expected errors per read, truncation of reads at the first instance of a QC score below 2, removal of phiX reads and trimming of 17 and 21 bases from the left side of the forward and reverse reads, respectively, to remove the primer sequences. Further data visualization was accomplished with the ggplot2 [68] package in R.

## Additional files

**Additional file 1: Figure S1.** Stacked barplot of the relative abundance of ASVs detected in each sample. ASVs are indicated by the colours displayed in the legend at the bottom of the figure. Reactor type and replicate are indicated in the horizontal axis labels in each facet (e.g. C1 is control reactor replicate 1, and H indicates the hydrogen-supplemented reactors). The text at the top of each facet indicates the sampling timepoint in days since reactor start-up. Samples with less than 1000 sequences were omitted from the figure. The taxonomic identity of each ASV may be found in the header for each facet in the ASV Catalogue, Additional file 2, and the 16S amplicon sequence for each ASV within the top 100 most abundant ASVs may be found in Additional file 5.

**Additional file 2.** ASV Catalogue. Multipage PDF format file with 100 facets corresponding to the top 100 most abundant ASVs. The ASV number and associated taxonomy are indicated by the text at the top of each facet. Datapoints in each figure indicate the abundance of the ASV in a reactor replicate sample at the time in days since reactor start-up. The solid line indicates the mean of control reactor abundances and the dashed line indicates that for the hydrogen-supplemented reactors, while symbol shape and fill indicate whether the datapoint corresponds to a reactor without H_2_ addition (control) or with (H_2_ supplemented), as indicated in the legend above each plot. Some ASVs indicate zero abundance across all timepoints, presumably because the elimination of samples with less than 1000 sequences resulted in the removal of spurious ASVs that comprised nearly the entirety of the few sequences present in the sample.

**Additional file 3.** Bioprocess data. Excel format spreadsheet of bioprocess data and factors corresponding to the microbial samples analysed. Columns are variables and factors, and rows are samples. Units are indicated in the column headers.

**Additional file 4.** Scatterplot matrices. Multipage PDF format file of pairwise scatterplots of bioprocess variables. The legend at the top of each page indicates the colouring of datapoints by phase of reactor operation or H_2_ supplementation (i.e. control vs. H_2_-supplemented). There are two plots for each colouring because some variables were not measured across all samples, and the two matrices with the lesser number of variables include all samples.

**Additional file 5.** FASTA sequences. FASTA format, 16S amplicon sequence file of the top 100 most abundant ASV sequences, with names in the FASTA headers corresponding to the names referenced in the article text and figures (e.g. ASV76 corresponds to *Methanobacterium* as discussed in the text and as indicated in the figures).

## Abbreviations

ASV: Amplicon Sequence Variant
BBU: Biological biogas upgrading
HRT: Hydraulic retention time
NMDS: Non-metric multidimensional scaling
OLR: Organic loading rate
PCA: principal components analysis
SRA: Sequence Read Archive
TAN: Total ammonium nitrogen
TCOD: Total chemical oxygen demand
VFA: Volatile fatty acid
