# Supplementary figures and images for: Effects of H2:CO2 ratio and H2 supply fluctuation on methane content and microbial community composition during in-situ biological biogas upgrading
Source: Biotechnol Biofuels. 2019 Apr 30;12:104. doi: 10.1186/s13068-019-1443-6 (PMC6489297; doi:10.1186/s13068-019-1443-6)

Day: 4

Day: 9

Day: 30

Day: 37

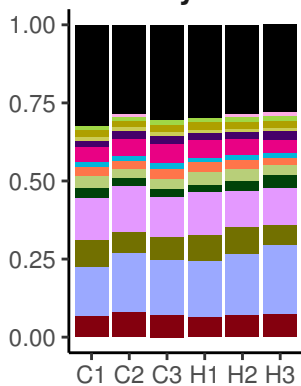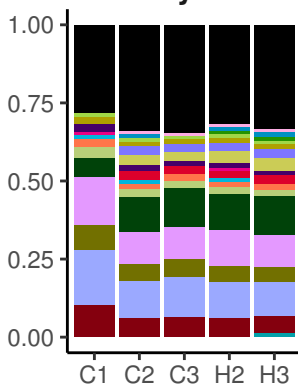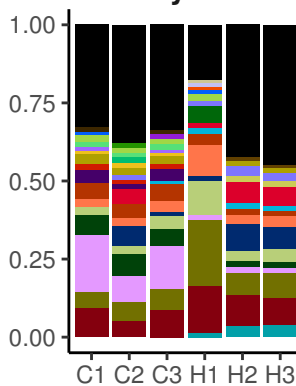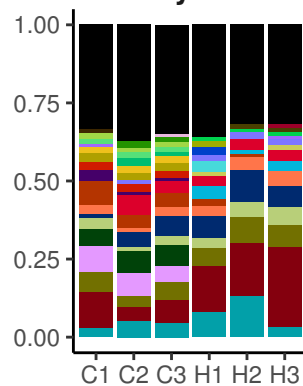

Day: 44

Day: 45

Day: 52

Day: 59

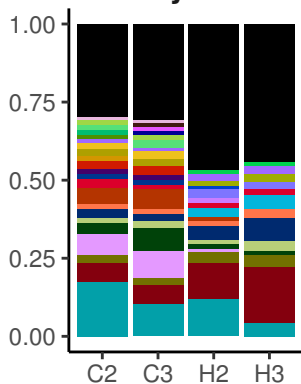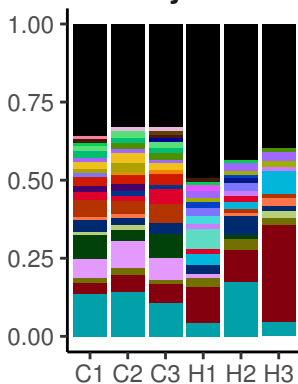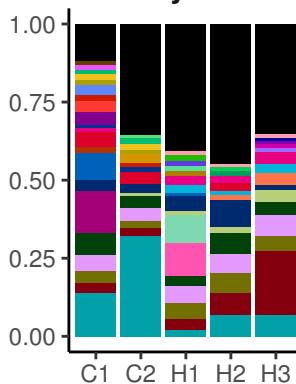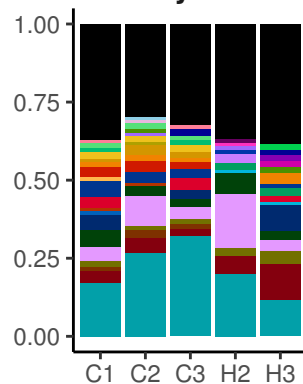

Day: 66

Day: 70

Day: 81

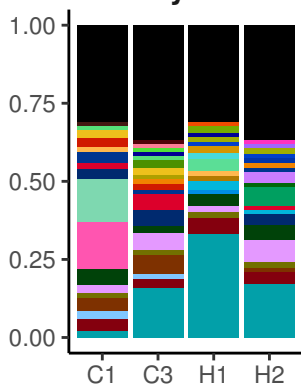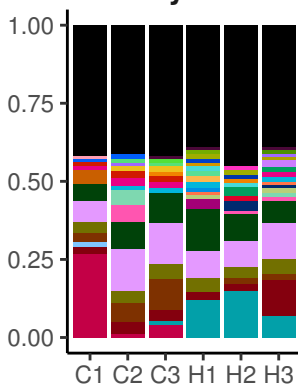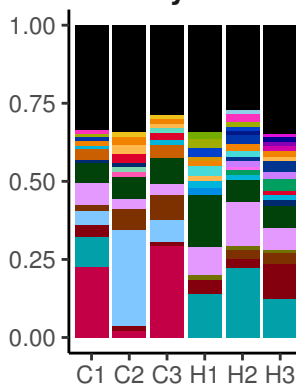

Reactor

ASV

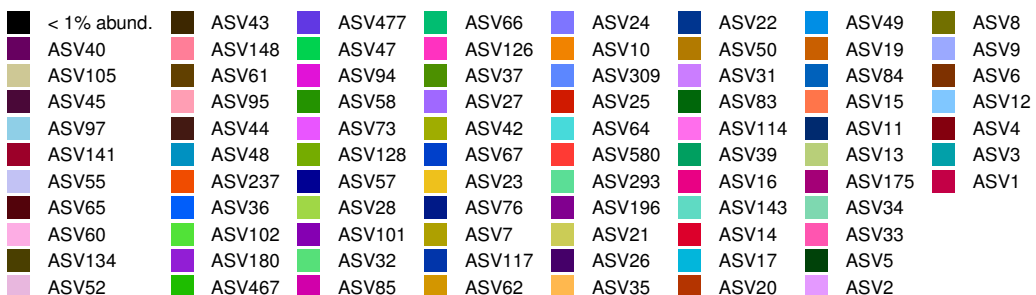

Supplement: Supplementary file 1 — Additional file 1: Figure S1. Stacked barplot of the relative abundance of ASVs detected in each sample. ASVs are indicated by the colours displayed in the legend at the bottom of the figure. Reactor type and replicate are indicated in the horizontal axis labels in each facet (e.g. C1 is control reactor replicate 1, and H indicates the hydrogen-supplemented reactors). The text at the top of each facet indicates the sampling timepoint in days since reactor start-up. Samples with less than 1000 sequences were omitted from the figure. The taxonomic identity of each ASV may be found in the header for each facet in the ASV Catalogue, Additional file 2, and the 16S amplicon sequence for each ASV within the top 100 most abundant ASVs may be found in Additional file 5. [file 13068_2019_1443_MOESM1_ESM.pdf]

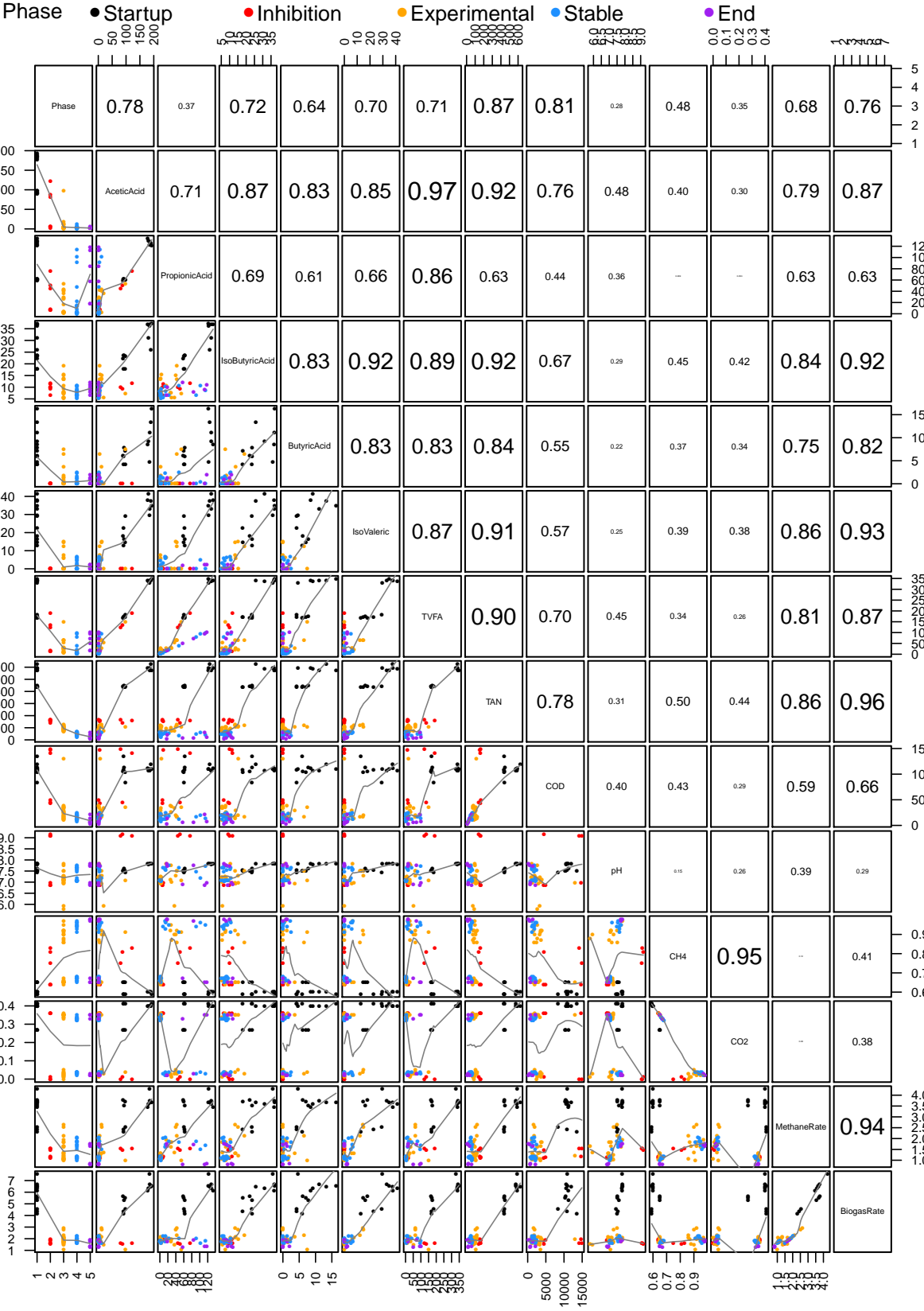

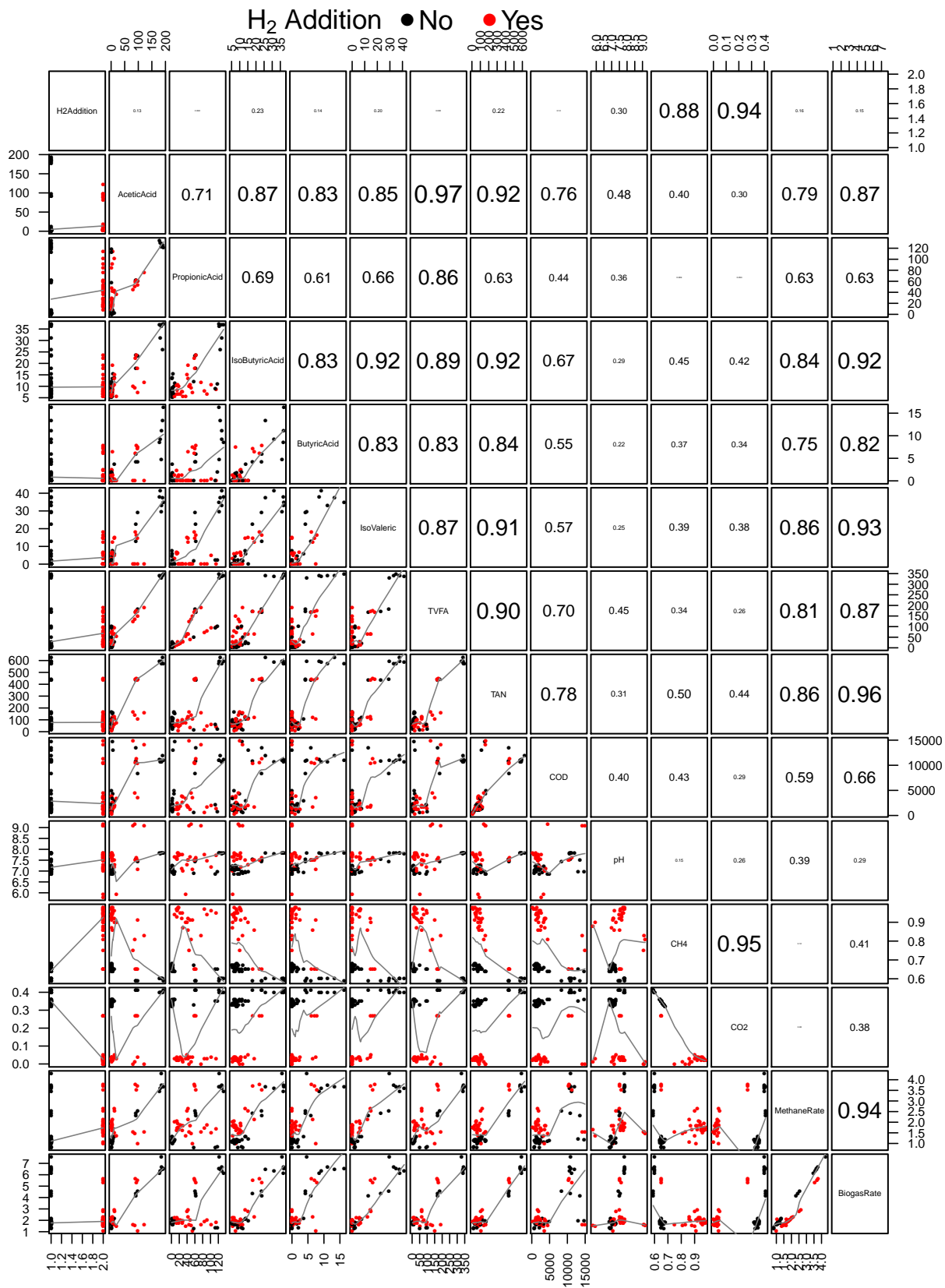

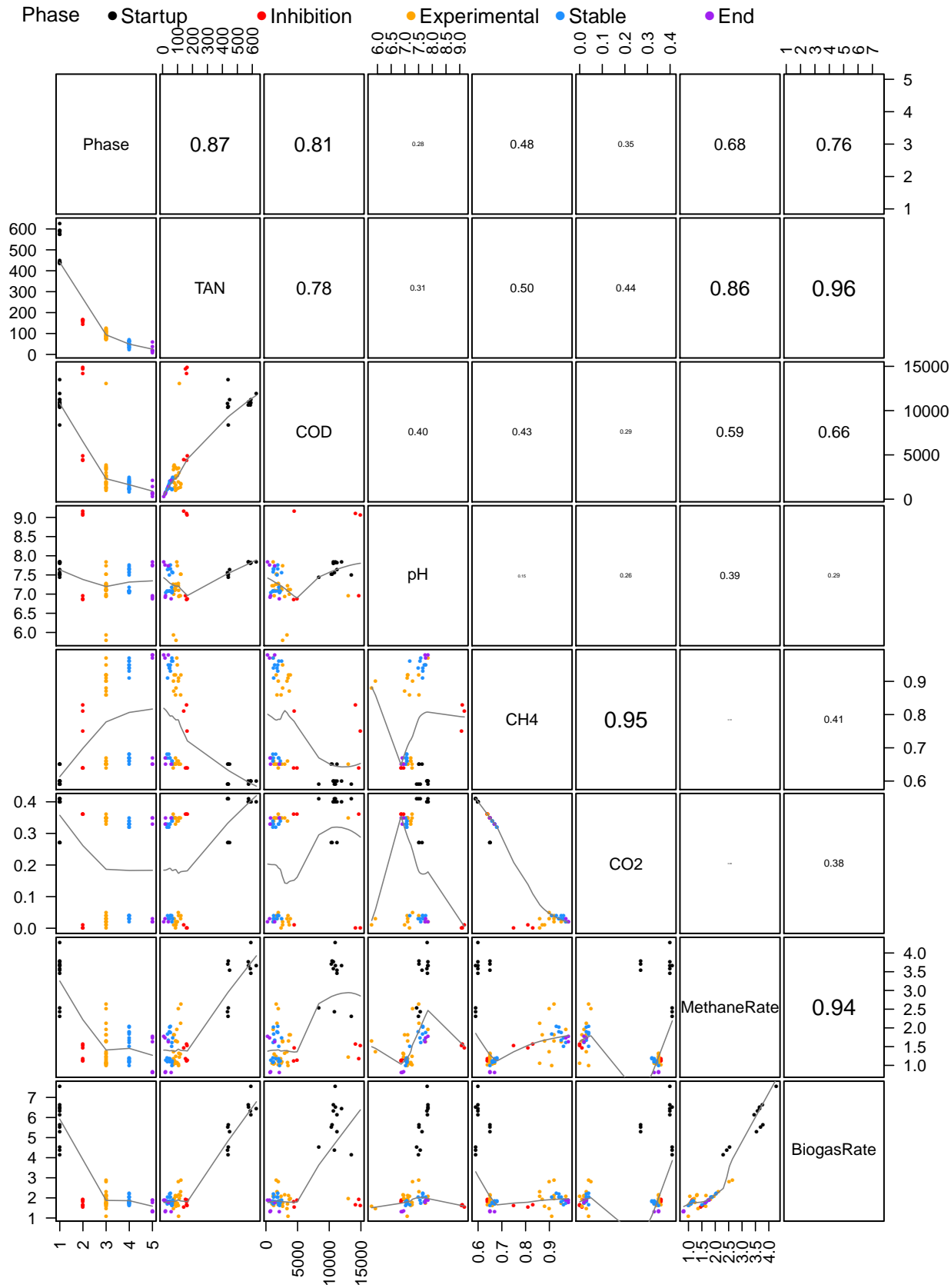

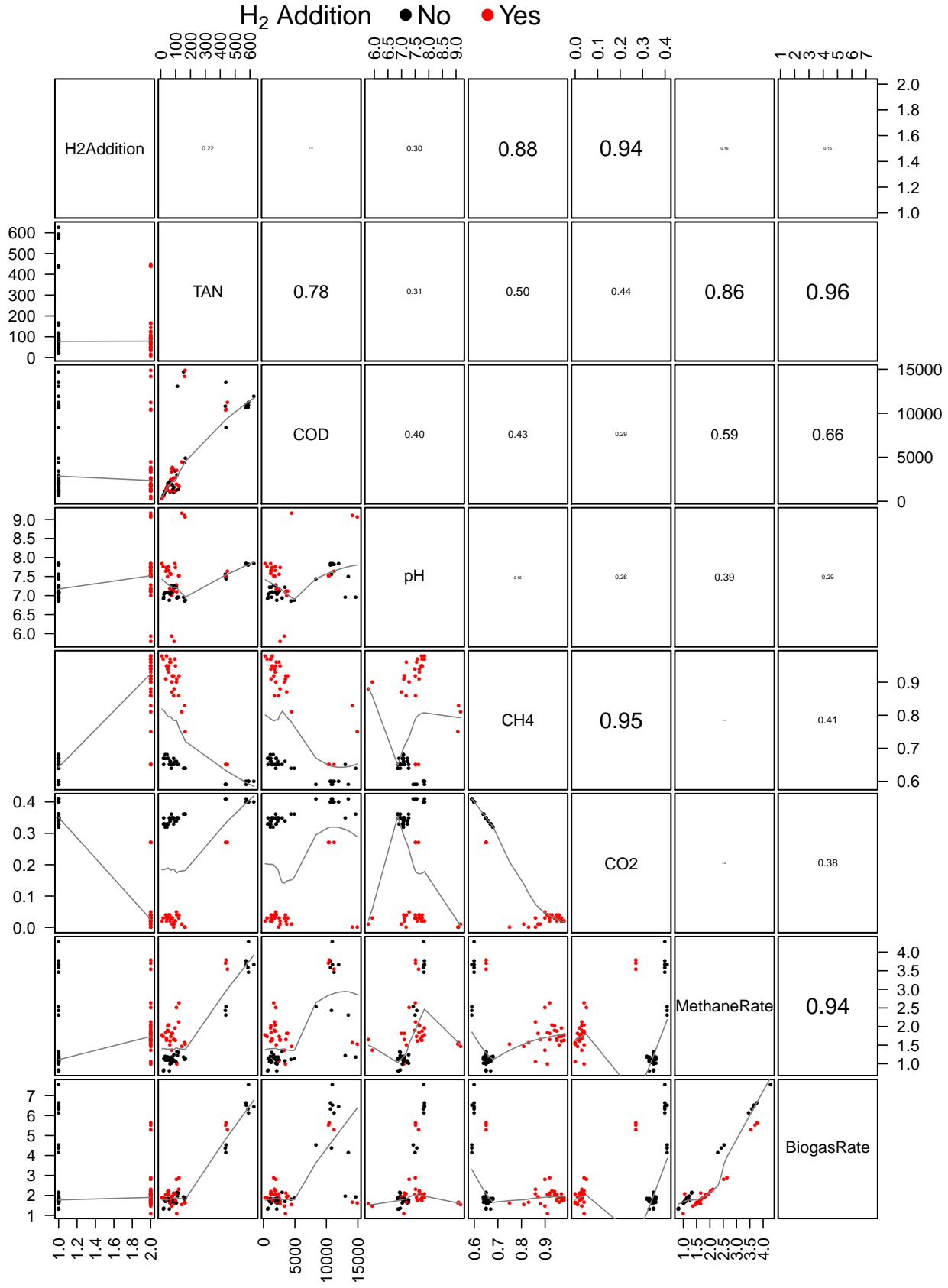

Supplement: Supplementary file 4 — Additional file 4. Scatterplot matrices. Multipage PDF format file of pairwise scatterplots of bioprocess variables. The legend at the top of each page indicates the colouring of datapoints by phase of reactor operation or H2 supplementation (i.e. control vs. H2-supplemented). There are two plots for each colouring because some variables were not measured across all samples, and the two matrices with the lesser number of variables include all samples. [file 13068_2019_1443_MOESM4_ESM.pdf]
