# Supplementary material for: Effects of H2:CO2 ratio and H2 supply fluctuation on methane content and microbial community composition during in-situ biological biogas upgrading
Source: Biotechnol Biofuels. 2019 Apr 30;12:104. doi: 10.1186/s13068-019-1443-6 (PMC6489297; doi:10.1186/s13068-019-1443-6)
Supplement: Supplementary file 2 — Additional file 2. ASV Catalogue. Multipage PDF format file with 100 facets corresponding to the top 100 most abundant ASVs. The ASV number and associated taxonomy are indicated by the text at the top of each facet. Datapoints in each figure indicate the abundance of the ASV in a reactor replicate sample at the time in days since reactor start-up. The solid line indicates the mean of control reactor abundances and the dashed line indicates that for the hydrogen-supplemented reactors, while symbol shape and fill indicate whether the datapoint corresponds to a reactor without H2 addition (control) or with (H2 supplemented), as indicated in the legend above each plot. Some ASVs indicate zero abundance across all timepoints, presumably because the elimination of samples with less than 1000 sequences resulted in the removal of spurious ASVs that comprised nearly the entirety of the few sequences present in the sample. [file 13068_2019_1443_MOESM2_ESM.pdf]

ASV1

Bacteria Proteobacteria Gammaproteobacteria Enterobacteriales
Enterobacteriaceae Escherichia/Shigella NA

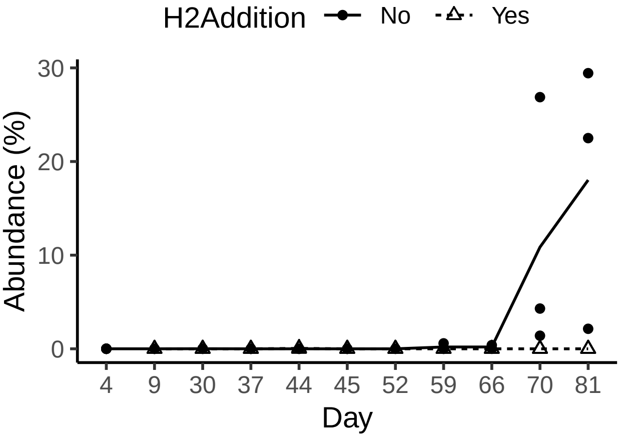

ASV4

Bacteria Bacteroidetes Bacteroidia Bacteroidales
Dysgonomonadaceae Fermentimonas caenicola

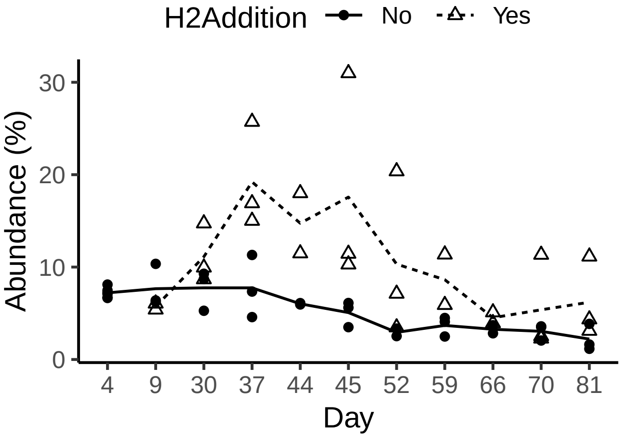

ASV2

Bacteria Bacteroidetes Bacteroidia Bacteroidales
Rikenellaceae DMER64 NA

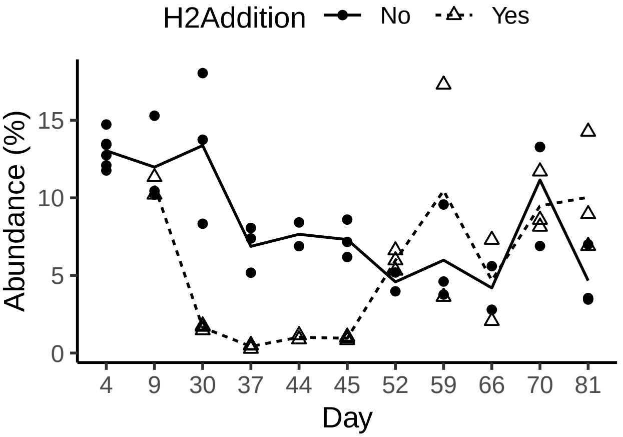

ASV5

Bacteria Cloacimonetes Cloacimonadia Cloacimonadales
Cloacimonadaceae Candidatus_Cloacimonas NA

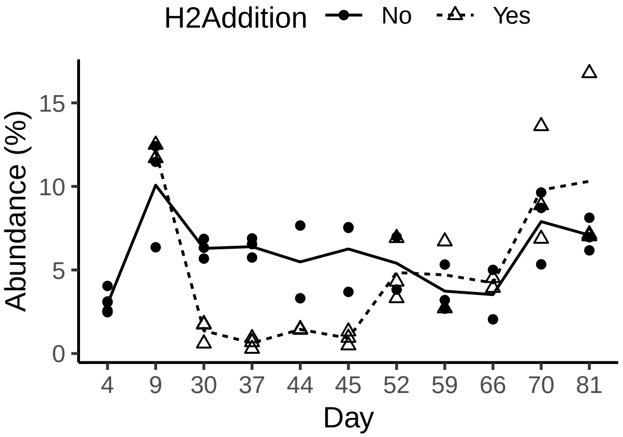

ASV3

Bacteria Firmicutes Bacilli Lactobacillales
Carnobacteriaceae Trichococcus NA

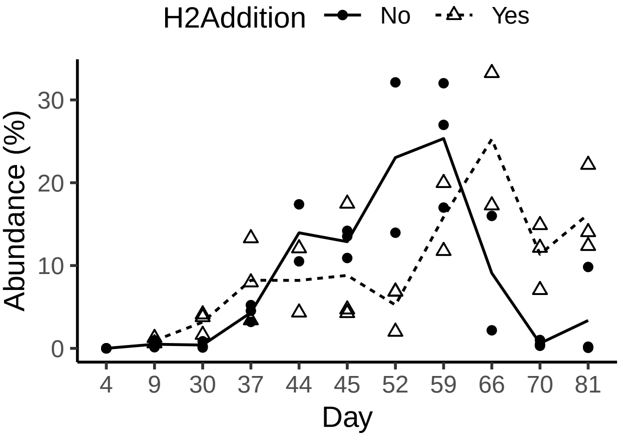

ASV6

Bacteria Spirochaetes Spirochaetia Spirochaetales
Spirochaetaceae NA NA

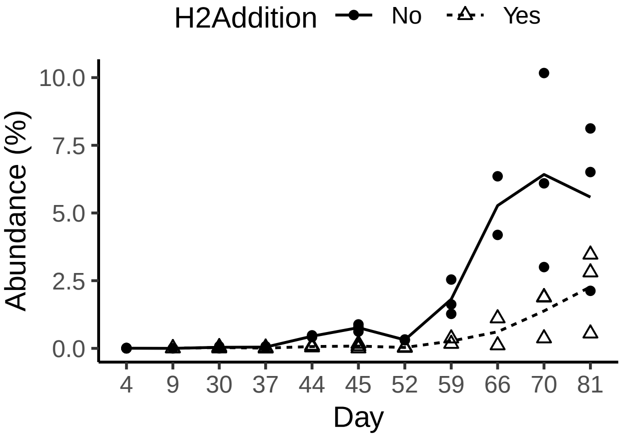

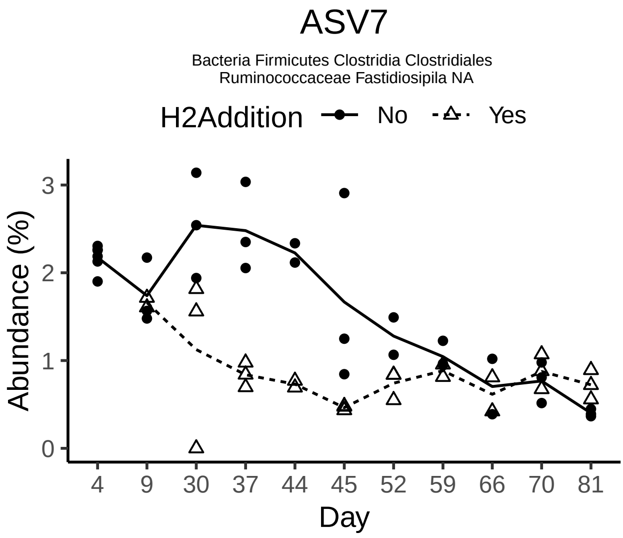
ASV7
Bacteria Firmicutes Clostridia Clostridiales
Ruminococcaceae Fastidiosipila NA
H2Addition No Yes
Abundance (%)
0
1
2
3
4 9 30 37 44 45 52 59 66 70 81
Day

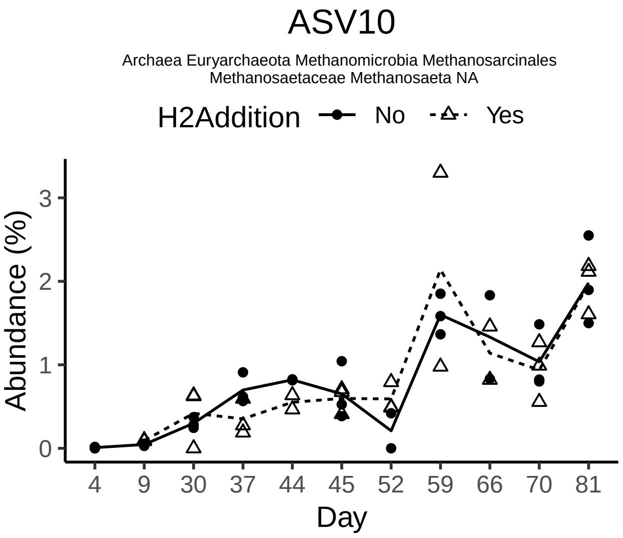
ASV10
Archaea Euryarchaeota Methanomicrobia Methanosarcinales
Methanosaetaceae Methanosaeta NA
H2Addition No Yes
Abundance (%)
0
1
2
3
4 9 30 37 44 45 52 59 66 70 81
Day

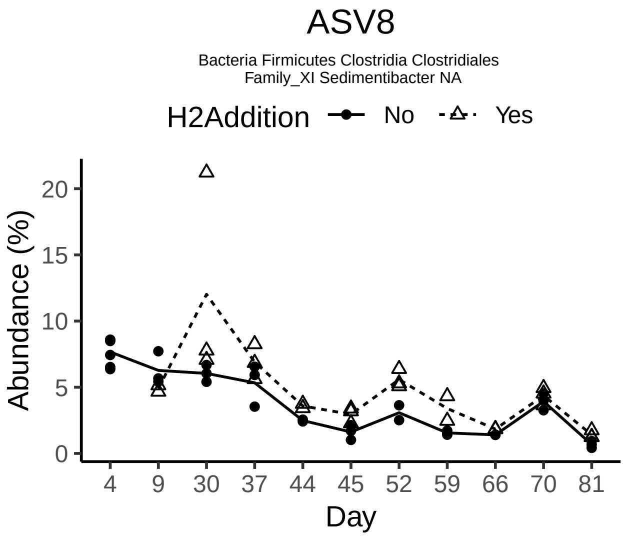
ASV8
Bacteria Firmicutes Clostridia Clostridiales
Family_XI Sedimentibacter NA
H2Addition No Yes
Abundance (%)
0
5
10
15
20
4 9 30 37 44 45 52 59 66 70 81
Day

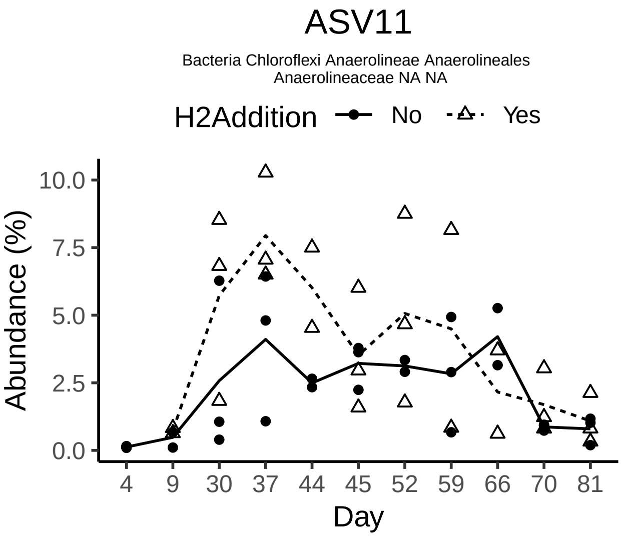
ASV11
Bacteria Chloroflexi Anaerolineae Anaerolineales
Anaerolineaceae NA NA
H2Addition No Yes
Abundance (%)
0.0
2.5
5.0
7.5
10.0
4 9 30 37 44 45 52 59 66 70 81
Day

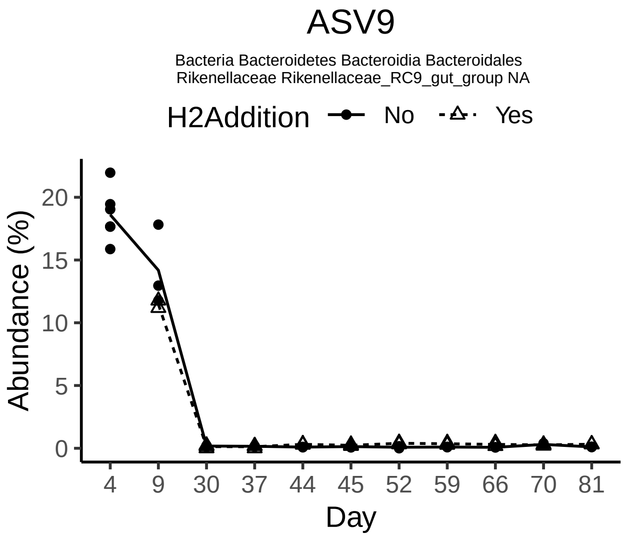
ASV9
Bacteria Bacteroidetes Bacteroidia Bacteroidales
Rikenellaceae Rikenellaceae_RC9_gut_group NA
H2Addition No Yes
Abundance (%)
0
5
10
15
20
4 9 30 37 44 45 52 59 66 70 81
Day

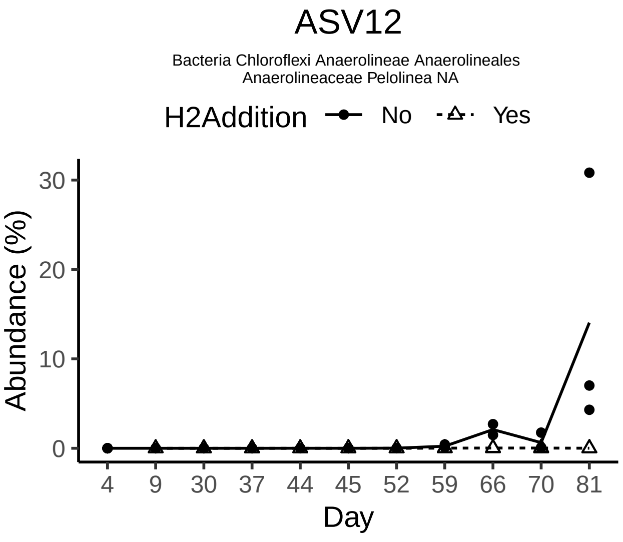
ASV12
Bacteria Chloroflexi Anaerolineae Anaerolineales
Anaerolineaceae Pelolinea NA
H2Addition No Yes
Abundance (%)
0
10
20
30
4 9 30 37 44 45 52 59 66 70 81
Day

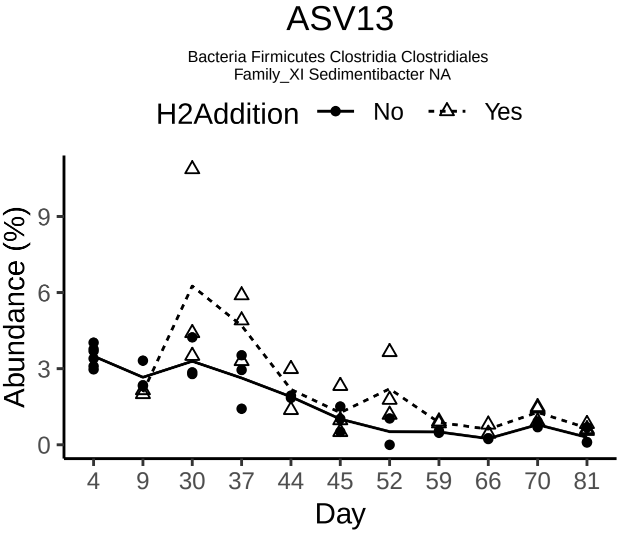
ASV13
Bacteria Firmicutes Clostridia Clostridiales
Family_XI Sedimentibacter NA
H2Addition No Yes
Abundance (%)
0
3
6
9
Day
4 9 30 37 44 45 52 59 66 70 81

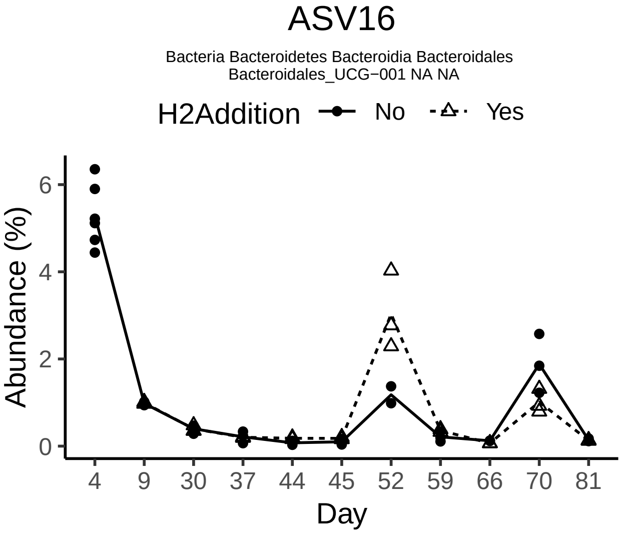
ASV16
Bacteria Bacteroidetes Bacteroidia Bacteroidales
Bacteroidales_UCG−001 NA NA
H2Addition No Yes
Abundance (%)
0
2
4
6
Day
4 9 30 37 44 45 52 59 66 70 81

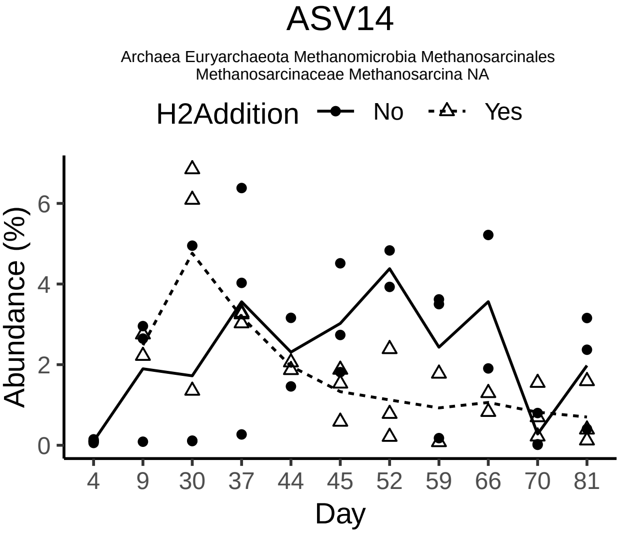
ASV14
Archaea Euryarchaeota Methanomicrobia Methanosarcinales
Methanosarcinaceae Methanosarcina NA
H2Addition No Yes
Abundance (%)
0
2
4
6
Day
4 9 30 37 44 45 52 59 66 70 81

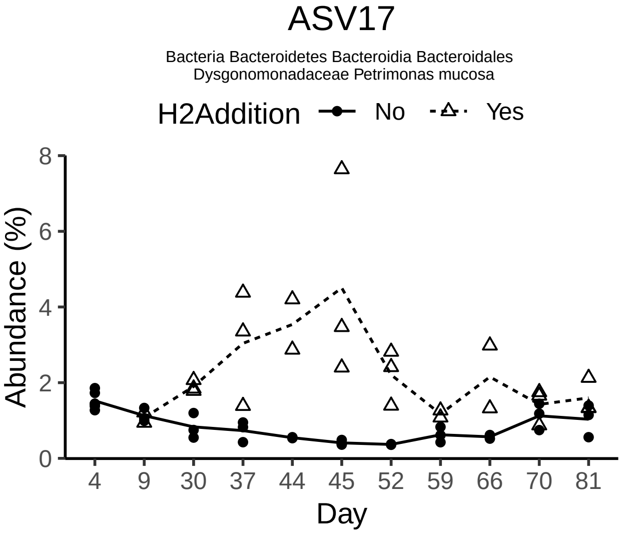
ASV17
Bacteria Bacteroidetes Bacteroidia Bacteroidales
Dysgonomonadaceae Petrimonas mucosa
H2Addition No Yes
Abundance (%)
0
2
4
6
8
Day
4 9 30 37 44 45 52 59 66 70 81

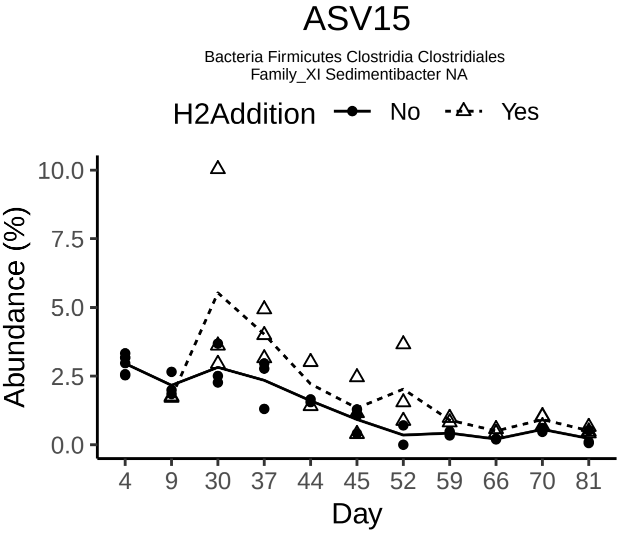
ASV15
Bacteria Firmicutes Clostridia Clostridiales
Family_XI Sedimentibacter NA
H2Addition No Yes
Abundance (%)
0.0
2.5
5.0
7.5
10.0
Day
4 9 30 37 44 45 52 59 66 70 81

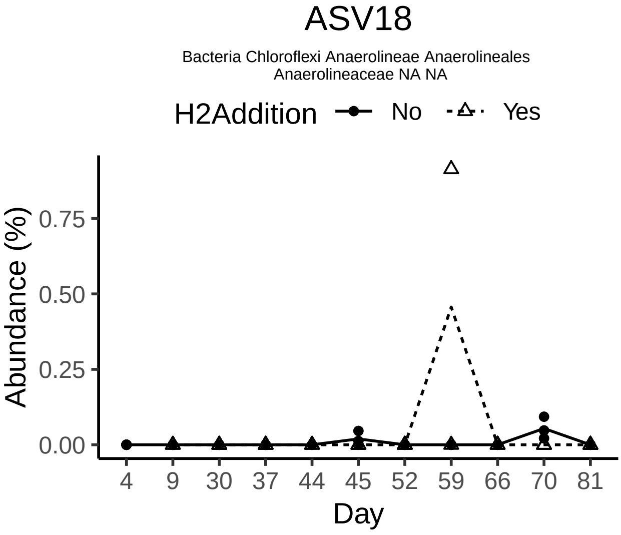
ASV18
Bacteria Chloroflexi Anaerolineae Anaerolineales
Anaerolineaceae NA NA
H2Addition No Yes
Abundance (%)
0.00
0.25
0.50
0.75
Day
4 9 30 37 44 45 52 59 66 70 81

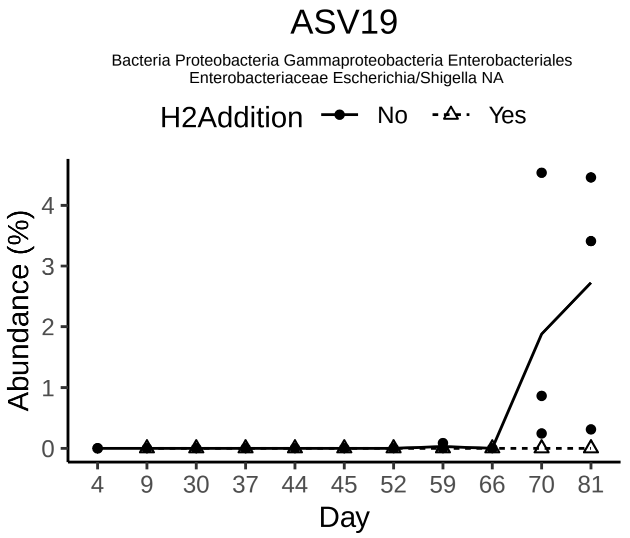
ASV19
Bacteria Proteobacteria Gammaproteobacteria Enterobacteriales
Enterobacteriaceae Escherichia/Shigella NA
H2Addition No Yes
Abundance (%)
0 1 2 3 4
4 9 30 37 44 45 52 59 66 70 81
Day

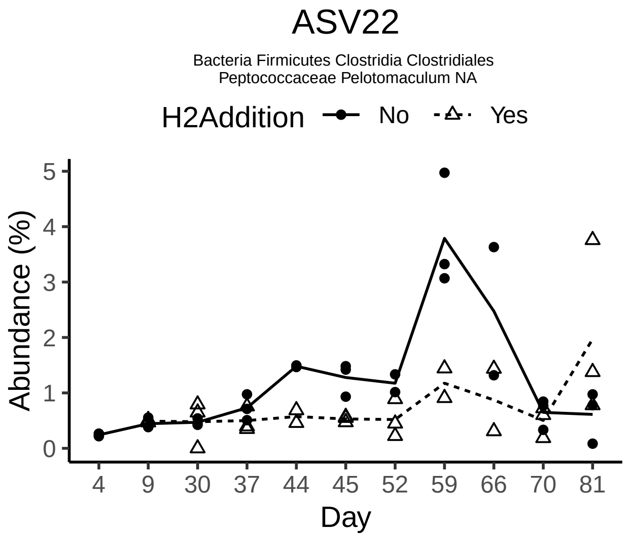
ASV22
Bacteria Firmicutes Clostridia Clostridiales
Peptococcaceae Pelotomaculum NA
H2Addition No Yes
Abundance (%)
0 1 2 3 4 5
4 9 30 37 44 45 52 59 66 70 81
Day

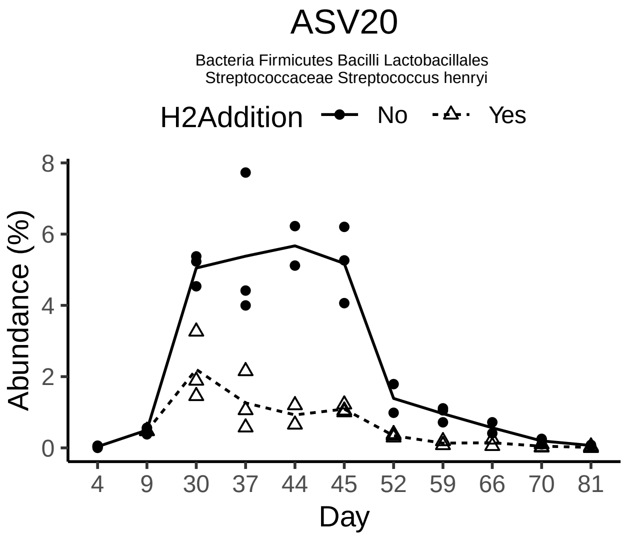
ASV20
Bacteria Firmicutes Bacilli Lactobacillales
Streptococcaceae Streptococcus henryi
H2Addition No Yes
Abundance (%)
0 2 4 6 8
4 9 30 37 44 45 52 59 66 70 81
Day

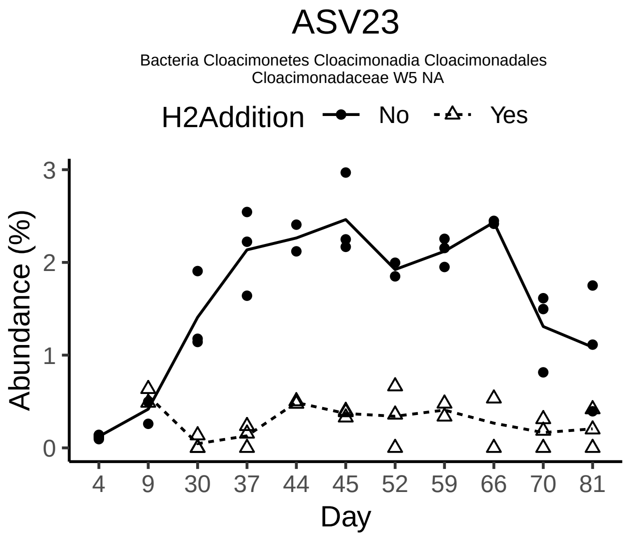
ASV23
Bacteria Cloacimonetes Cloacimonadia Cloacimonadales
Cloacimonadaceae W5 NA
H2Addition No Yes
Abundance (%)
0 1 2 3
4 9 30 37 44 45 52 59 66 70 81
Day

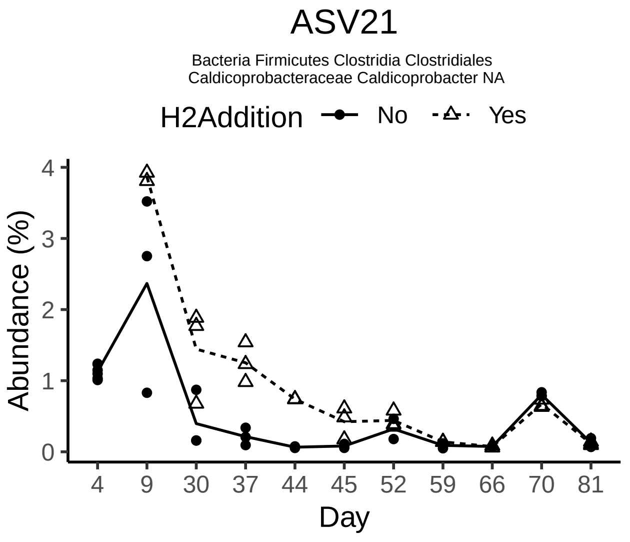
ASV21
Bacteria Firmicutes Clostridia Clostridiales
Caldicoprobacteraceae Caldicoprobacter NA
H2Addition No Yes
Abundance (%)
0 1 2 3 4
4 9 30 37 44 45 52 59 66 70 81
Day

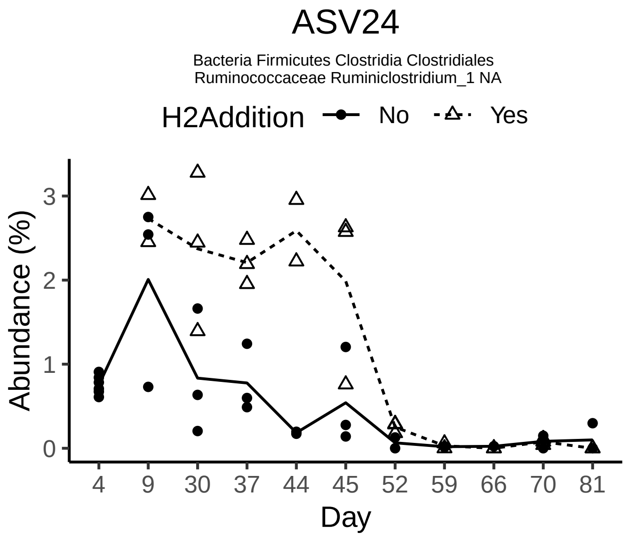
ASV24
Bacteria Firmicutes Clostridia Clostridiales
Ruminococcaceae Ruminiclostridium_1 NA
H2Addition No Yes
Abundance (%)
0 1 2 3
4 9 30 37 44 45 52 59 66 70 81
Day

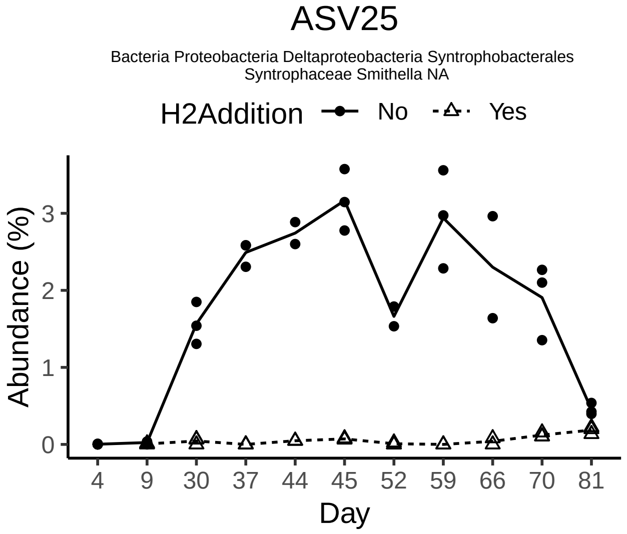
ASV25
Bacteria Proteobacteria Deltaproteobacteria Syntrophobacterales
Syntrophaceae Smithella NA
H2Addition
No
Yes
Abundance (%)
0
1
2
3
4
9
30
37
44
45
52
59
66
70
81
Day

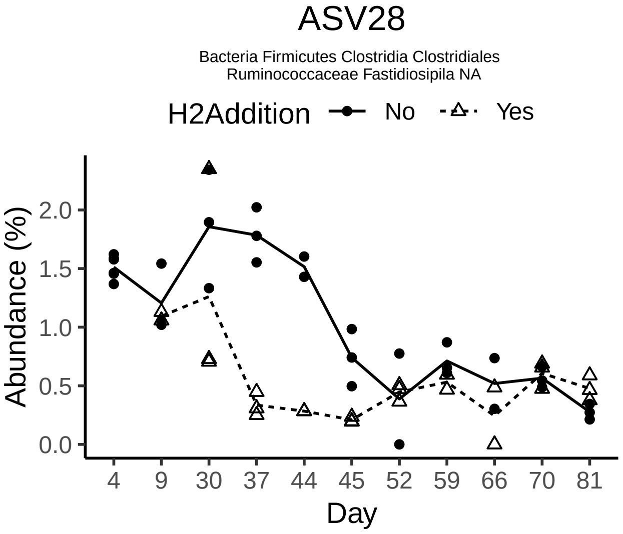
ASV28
Bacteria Firmicutes Clostridia Clostridiales
Ruminococcaceae Fastidiosipila NA
H2Addition
No
Yes
Abundance (%)
0.0
0.5
1.0
1.5
2.0
4
9
30
37
44
45
52
59
66
70
81
Day

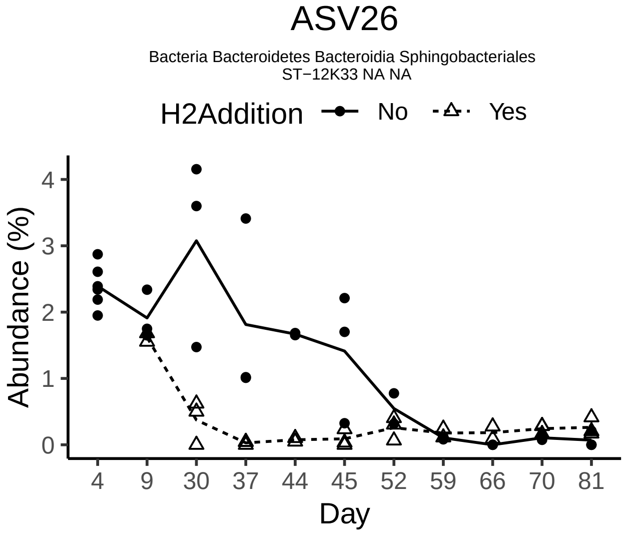
ASV26
Bacteria Bacteroidetes Bacteroidia Sphingobacteriales
ST−12K33 NA NA
H2Addition
No
Yes
Abundance (%)
0
1
2
3
4
4
9
30
37
44
45
52
59
66
70
81
Day

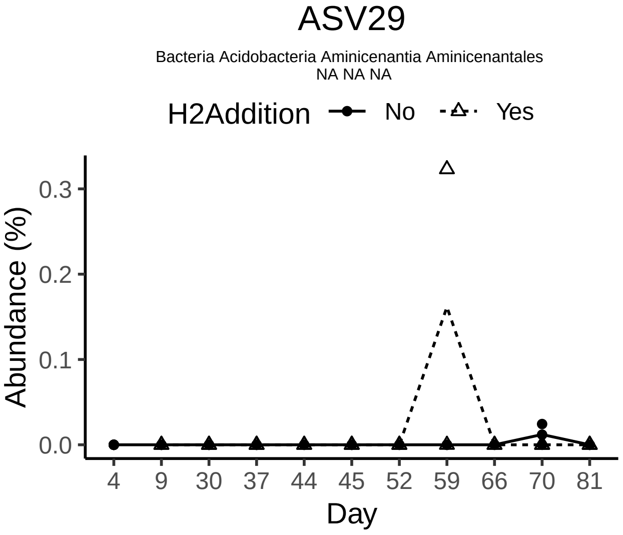
ASV29
Bacteria Acidobacteria Aminicenantia Aminicenantales
NA NA NA
H2Addition
No
Yes
Abundance (%)
0.0
0.1
0.2
0.3
4
9
30
37
44
45
52
59
66
70
81
Day

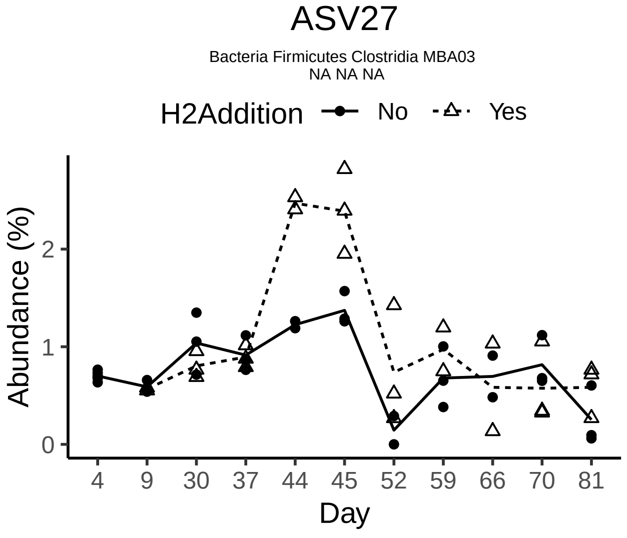
ASV27
Bacteria Firmicutes Clostridia MBA03
NA NA NA
H2Addition
No
Yes
Abundance (%)
0
1
2
4
9
30
37
44
45
52
59
66
70
81
Day

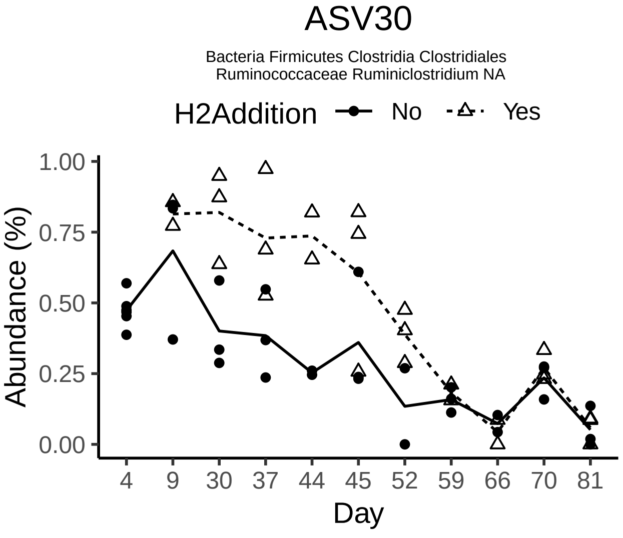
ASV30
Bacteria Firmicutes Clostridia Clostridiales
Ruminococcaceae Ruminiclostridium NA
H2Addition
No
Yes
Abundance (%)
0.00
0.25
0.50
0.75
1.00
4
9
30
37
44
45
52
59
66
70
81
Day

ASV31

Bacteria Firmicutes Clostridia DTU014
NA NA NA

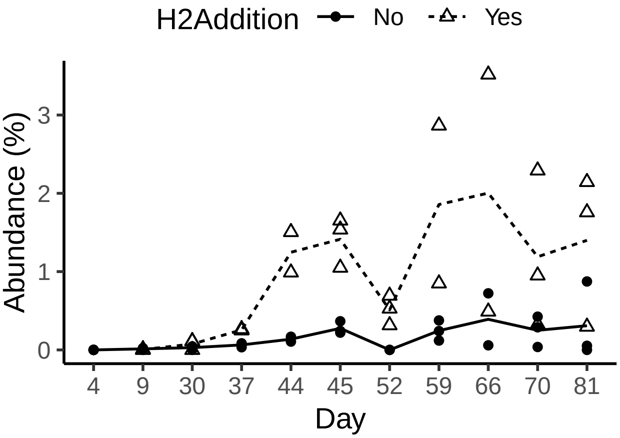

ASV34

Bacteria Proteobacteria Gammaproteobacteria Enterobacteriales
Enterobacteriaceae Enterobacter NA

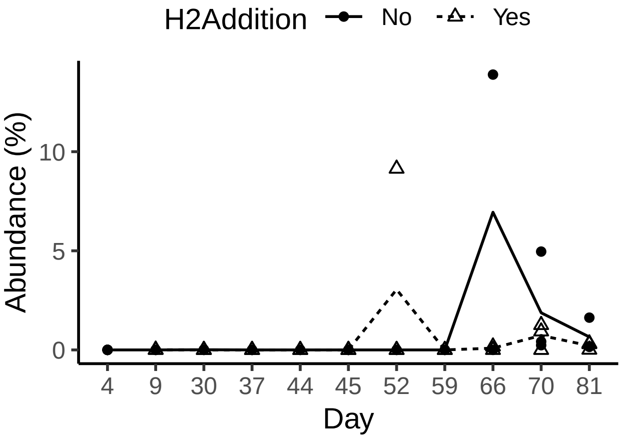

ASV32

Bacteria Cloacimonetes Cloacimonadia Cloacimonadales
Cloacimonadaceae W5 NA

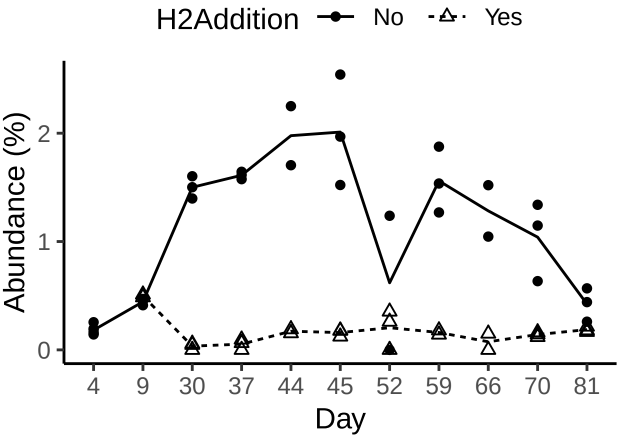

ASV35

Bacteria Actinobacteria Actinobacteria Propionibacteriales
Propionibacteriaceae NA NA

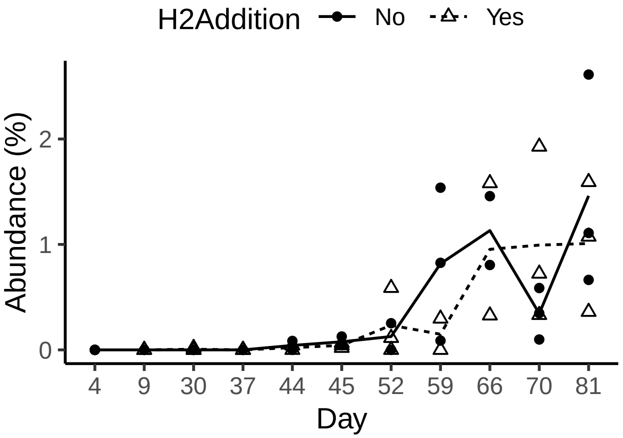

ASV33

Bacteria Proteobacteria Gammaproteobacteria Enterobacteriales
Enterobacteriaceae Enterobacter NA

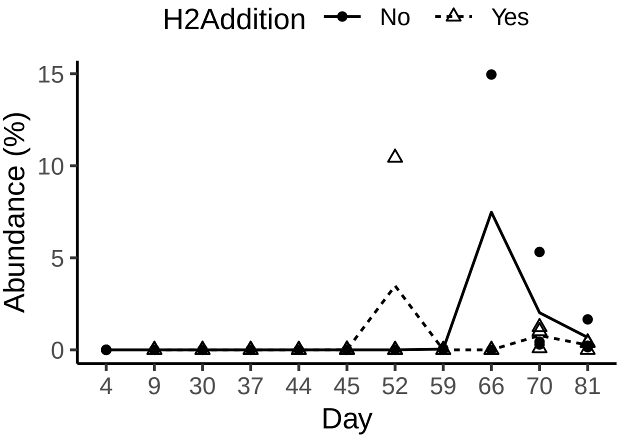

ASV36

Bacteria Firmicutes Clostridia MBA03
NA NA NA

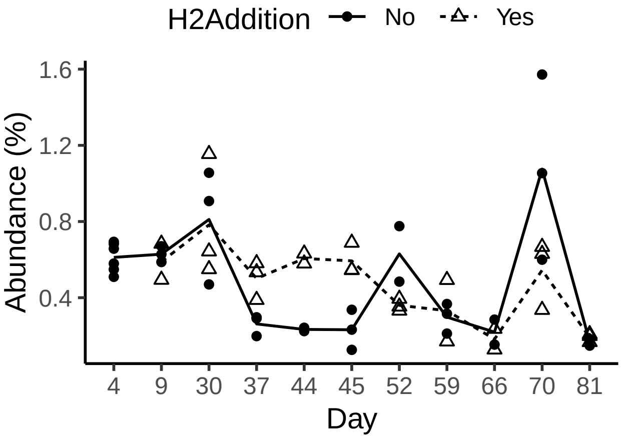

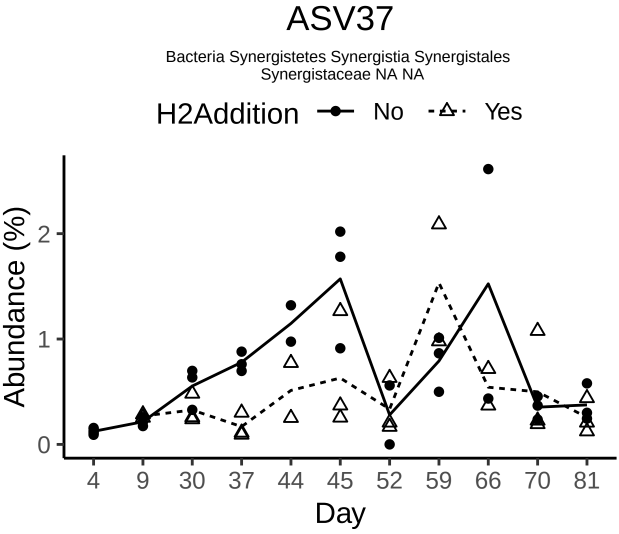
ASV37
Bacteria Synergistetes Synergistia Synergistales
Synergistaceae NA NA
H2Addition No Yes
Abundance (%)
0
1
2
4 9 30 37 44 45 52 59 66 70 81
Day

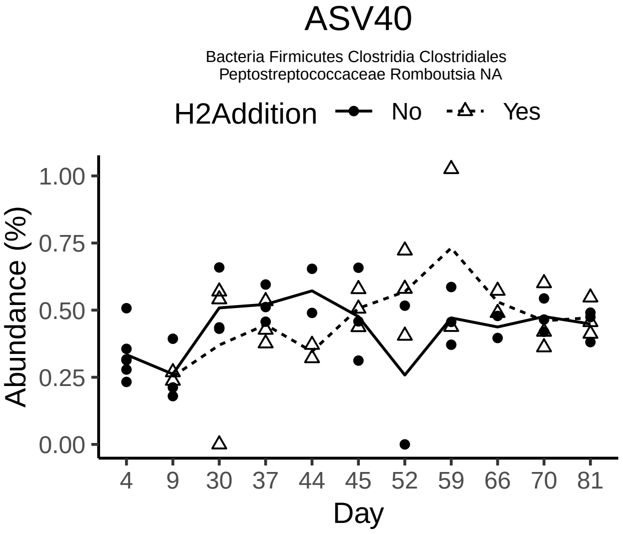
ASV40
Bacteria Firmicutes Clostridia Clostridiales
Peptostreptococcaceae Romboutsia NA
H2Addition No Yes
Abundance (%)
0.00
0.25
0.50
0.75
1.00
4 9 30 37 44 45 52 59 66 70 81
Day

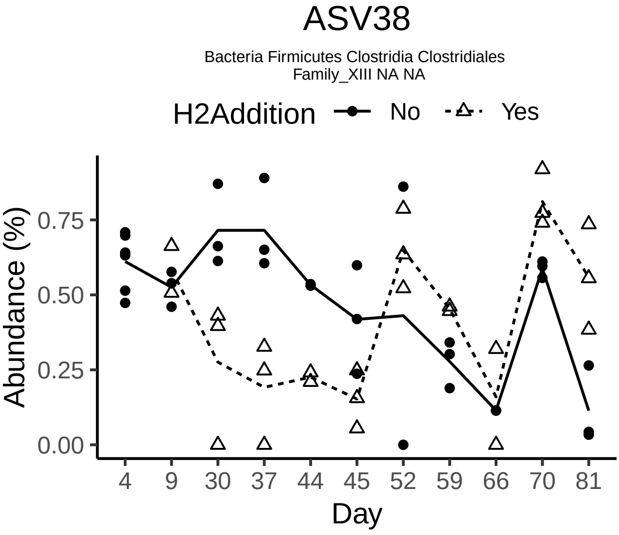
ASV38
Bacteria Firmicutes Clostridia Clostridiales
Family_XIII NA NA
H2Addition No Yes
Abundance (%)
0.00
0.25
0.50
0.75
4 9 30 37 44 45 52 59 66 70 81
Day

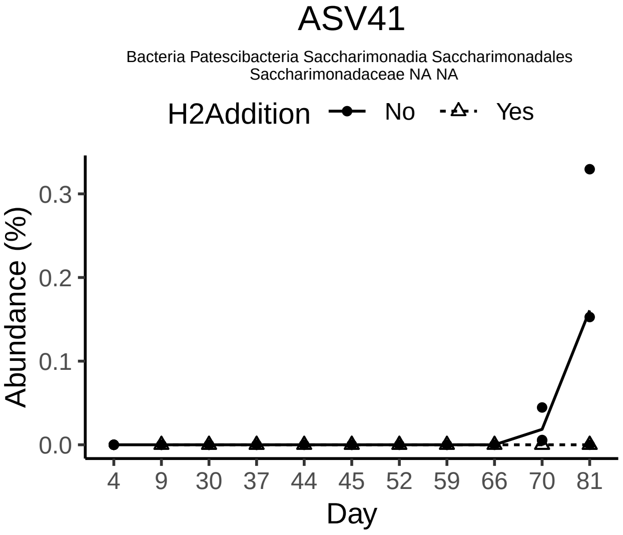
ASV41
Bacteria Patescibacteria Saccharimonadia Saccharimonadales
Saccharimonadaceae NA NA
H2Addition No Yes
Abundance (%)
0.0
0.1
0.2
0.3
4 9 30 37 44 45 52 59 66 70 81
Day

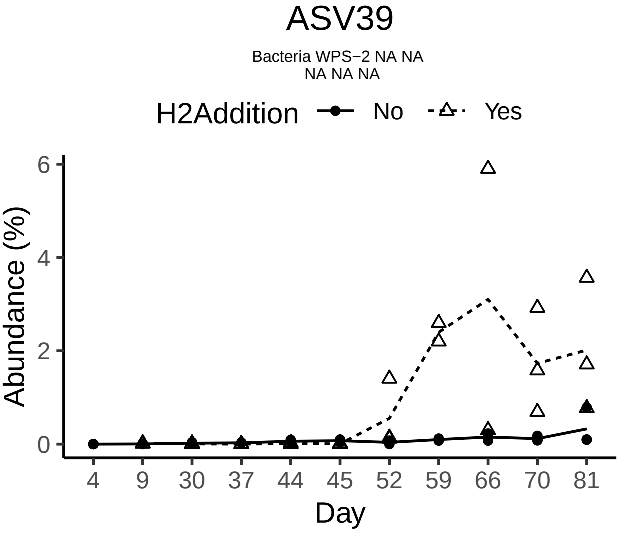
ASV39
Bacteria WPS−2 NA NA
NA NA NA
H2Addition No Yes
Abundance (%)
0
2
4
6
4 9 30 37 44 45 52 59 66 70 81
Day

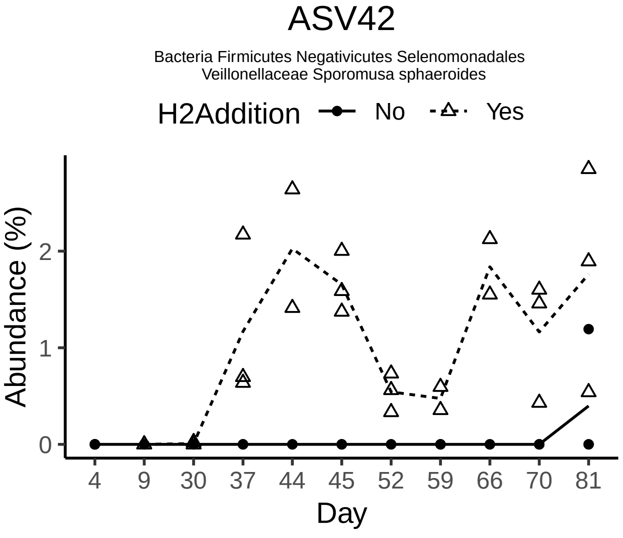
ASV42
Bacteria Firmicutes Negativicutes Selenomonadales
Veillonellaceae Sporomusa sphaeroides
H2Addition No Yes
Abundance (%)
0
1
2
4 9 30 37 44 45 52 59 66 70 81
Day

ASV43

Bacteria Firmicutes Clostridia MBA03
NA NA NA

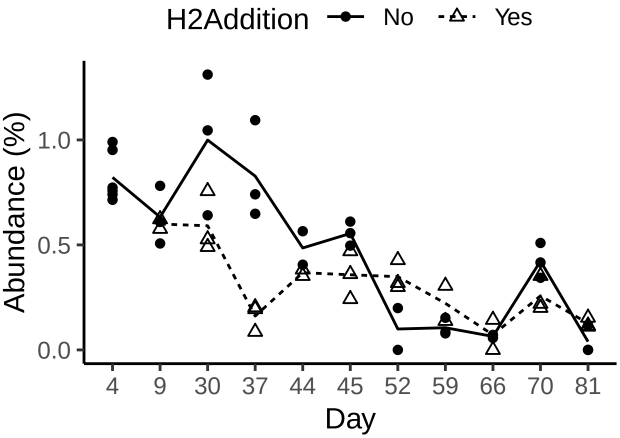

ASV46

Bacteria Firmicutes Clostridia MBA03
NA NA NA

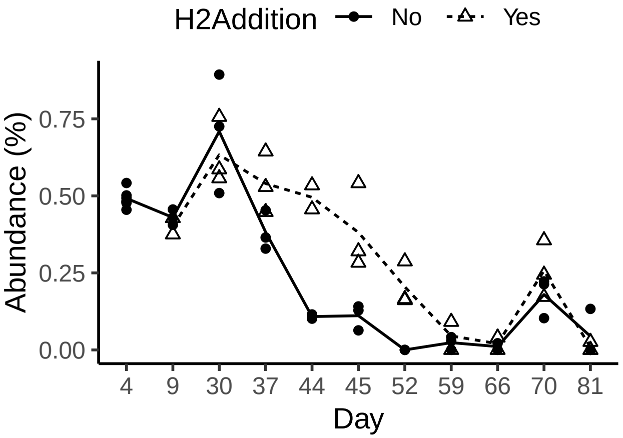

ASV44

Bacteria Proteobacteria Deltaproteobacteria Desulfovibrionales
Desulfovibrionaceae Desulfovibrio NA

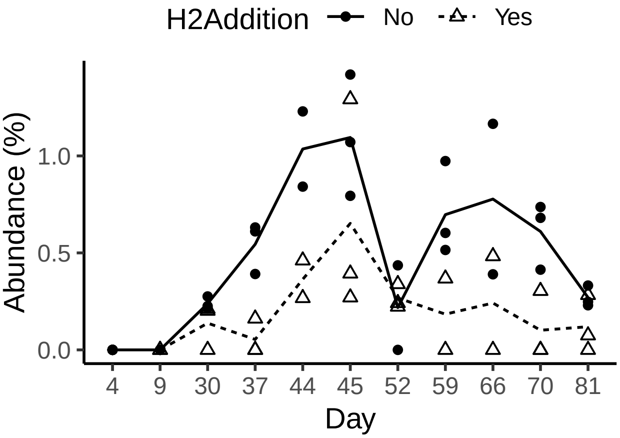

ASV47

Bacteria Bacteroidetes Bacteroidia Bacteroidales
Prolixibacteraceae NA NA

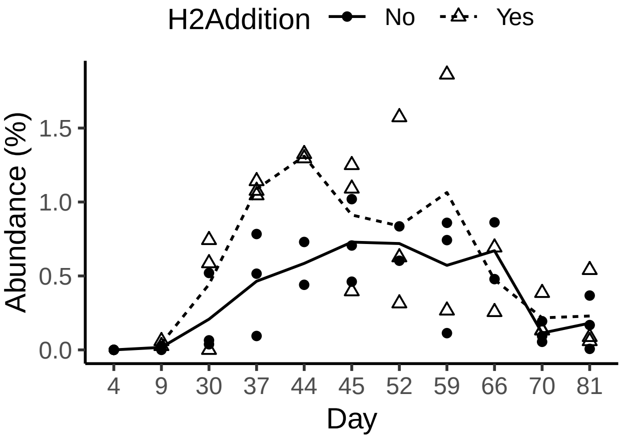

ASV45

Bacteria Firmicutes Clostridia Clostridiales
Syntrophomonadaceae Syntrophomonas NA

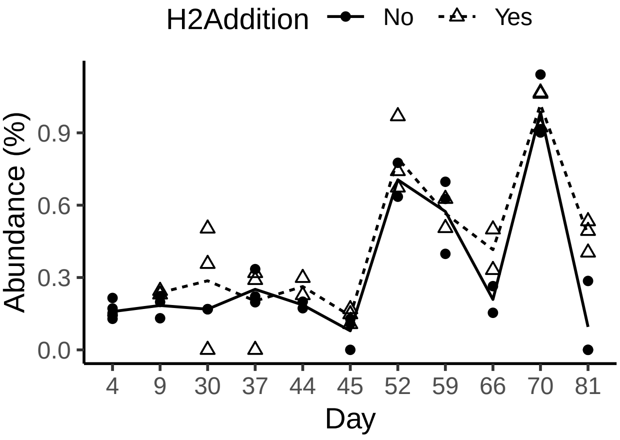

ASV48

Bacteria Firmicutes Clostridia Clostridiales
Lachnospiraceae Herbinix NA

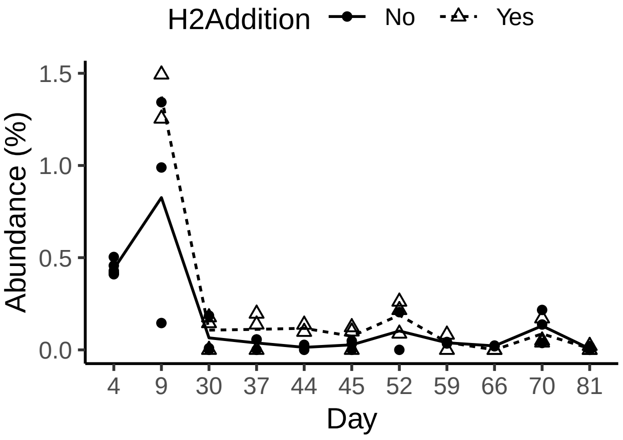

ASV49

Bacteria Firmicutes Clostridia Clostridiales
Heliobacteriaceae Hydrogenispora NA

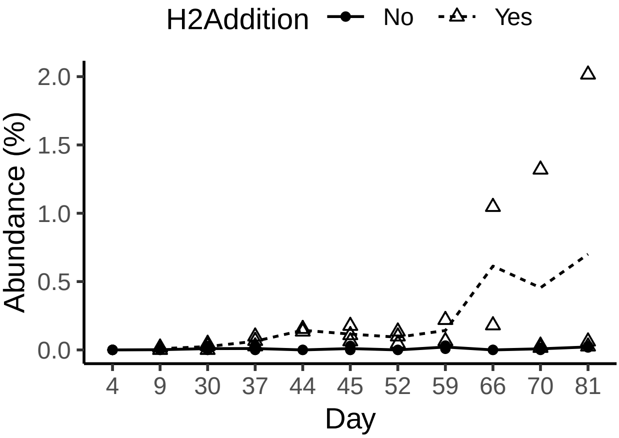

ASV52

Bacteria Cloacimonetes Cloacimonadia Cloacimonadales
Cloacimonadaceae W5 NA

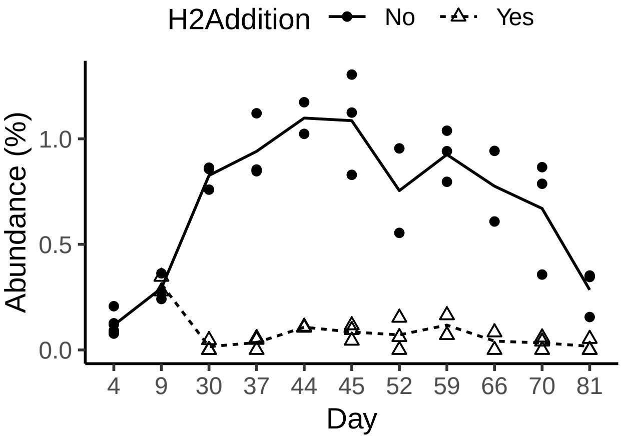

ASV50

Bacteria Bacteroidetes Bacteroidia Bacteroidales
Dysgonomonadaceae Dysgonomonas oryzarvi

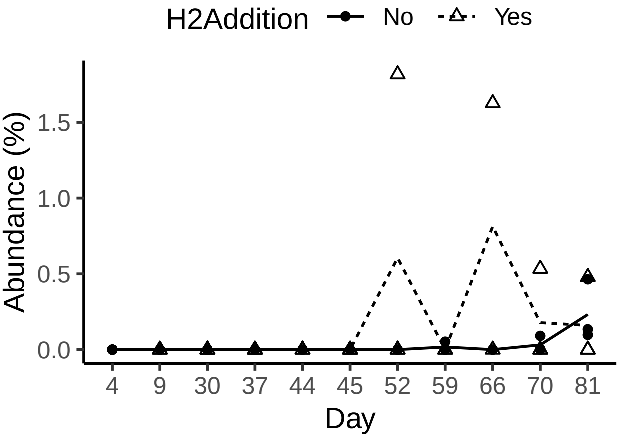

ASV53

Bacteria Chloroflexi Anaerolineae Anaerolineales
Anaerolineaceae NA NA

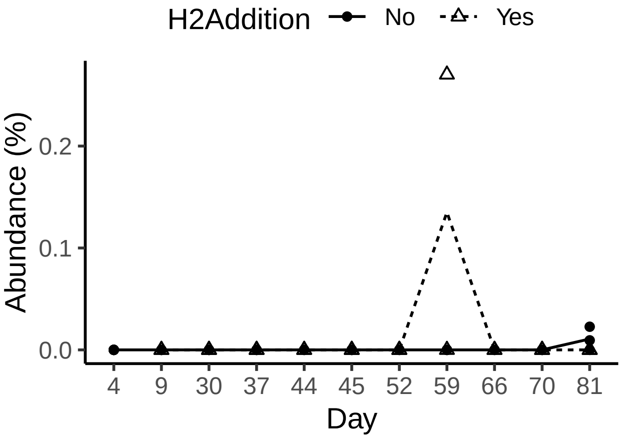

ASV51

Bacteria Firmicutes Clostridia Clostridiales
Ruminococcaceae NA NA

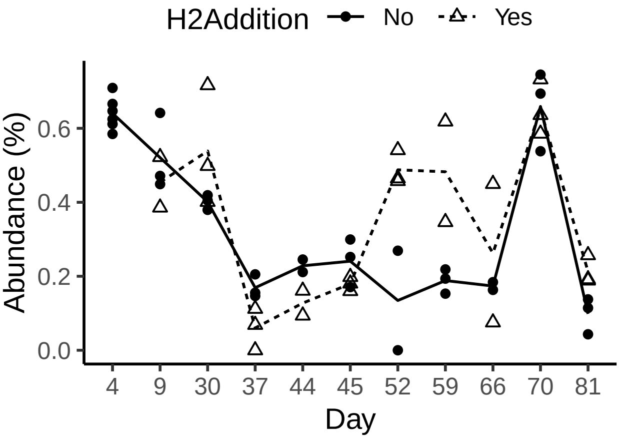

ASV54

Bacteria Firmicutes Clostridia Clostridiales
Syntrophomonadaceae Syntrophomonas NA

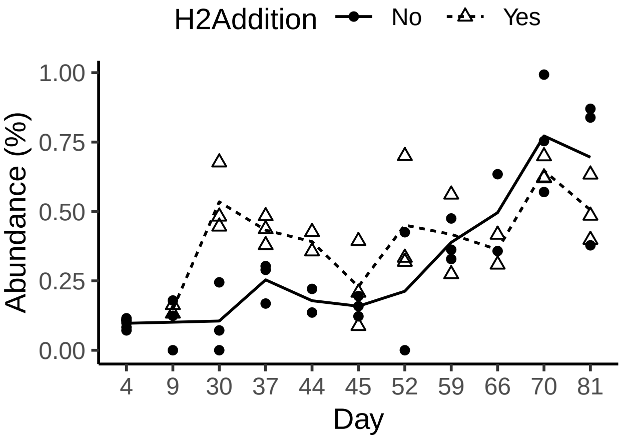

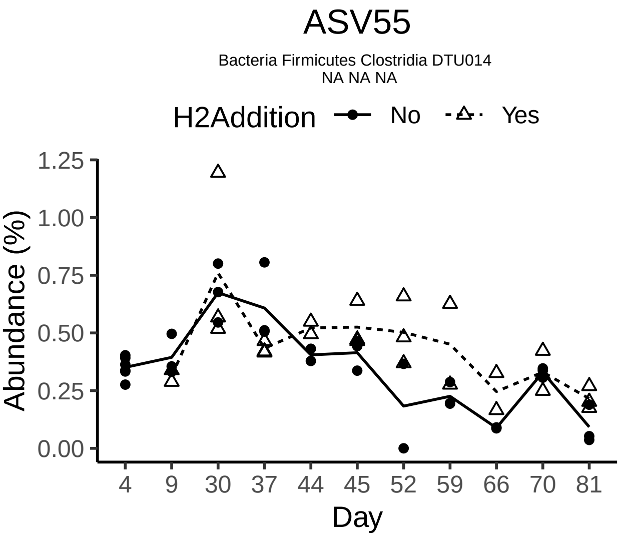
ASV55
Bacteria Firmicutes Clostridia DTU014
NA NA NA
H2Addition
No
Yes
Abundance (%)
0.00
0.25
0.50
0.75
1.00
1.25
4 9 30 37 44 45 52 59 66 70 81
Day

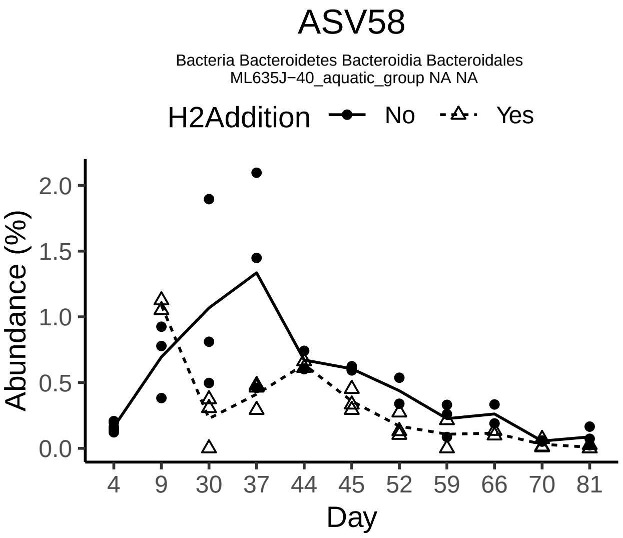
ASV58
Bacteria Bacteroidetes Bacteroidia Bacteroidales
ML635J−40_aquatic_group NA NA
H2Addition
No
Yes
Abundance (%)
0.0
0.5
1.0
1.5
2.0
4 9 30 37 44 45 52 59 66 70 81
Day

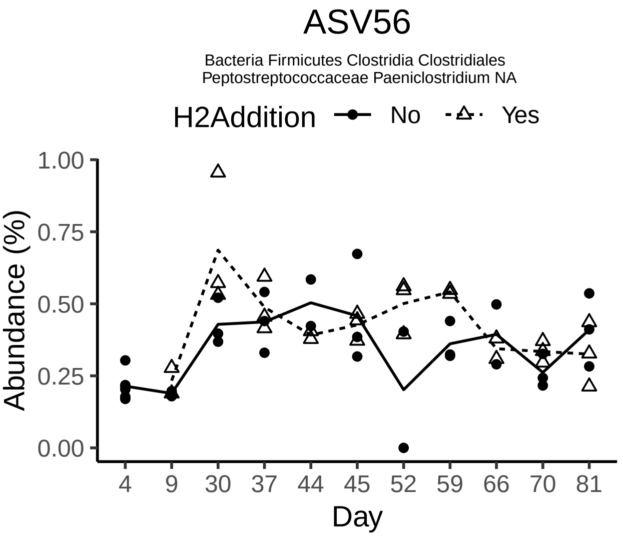
ASV56
Bacteria Firmicutes Clostridia Clostridiales
Peptostreptococcaceae Paeniclostridium NA
H2Addition
No
Yes
Abundance (%)
0.00
0.25
0.50
0.75
1.00
4 9 30 37 44 45 52 59 66 70 81
Day

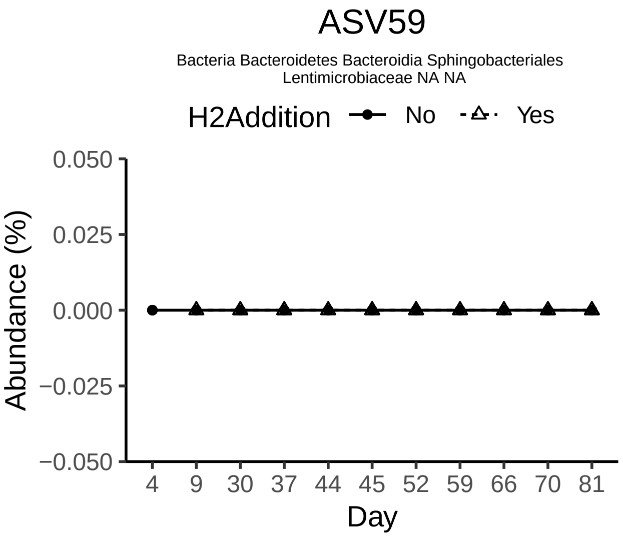
ASV59
Bacteria Bacteroidetes Bacteroidia Sphingobacteriales
Lentimicrobiaceae NA NA
H2Addition
No
Yes
Abundance (%)
−0.050
−0.025
0.000
0.025
0.050
4 9 30 37 44 45 52 59 66 70 81
Day

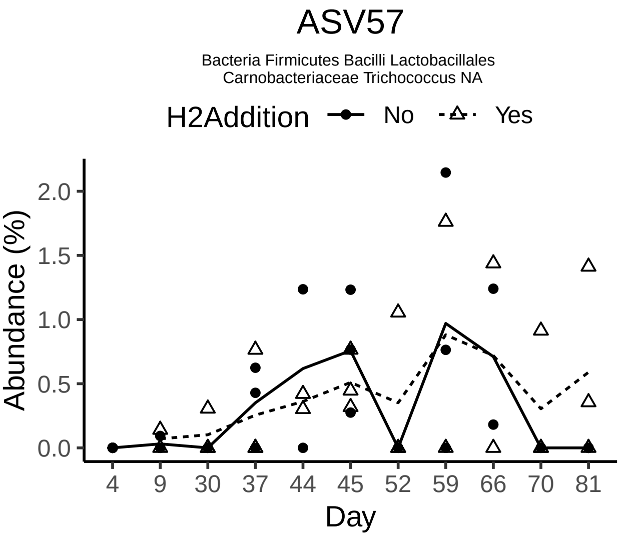
ASV57
Bacteria Firmicutes Bacilli Lactobacillales
Carnobacteriaceae Trichococcus NA
H2Addition
No
Yes
Abundance (%)
0.0
0.5
1.0
1.5
2.0
4 9 30 37 44 45 52 59 66 70 81
Day

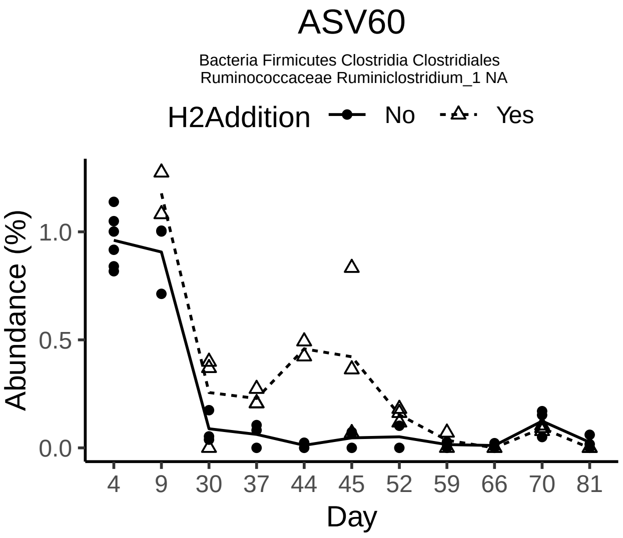
ASV60
Bacteria Firmicutes Clostridia Clostridiales
Ruminococcaceae Ruminiclostridium_1 NA
H2Addition
No
Yes
Abundance (%)
0.0
0.5
1.0
4 9 30 37 44 45 52 59 66 70 81
Day

ASV61

Archaea Euryarchaeota Methanomicrobia Methanosarcinales
Methanosarcinaceae Methanosarcina NA

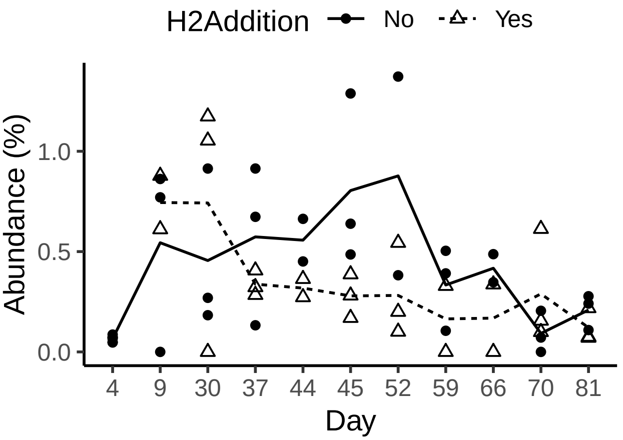

ASV64

Bacteria Firmicutes Negativicutes Selenomonadales
Veillonellaceae Sporomusa NA

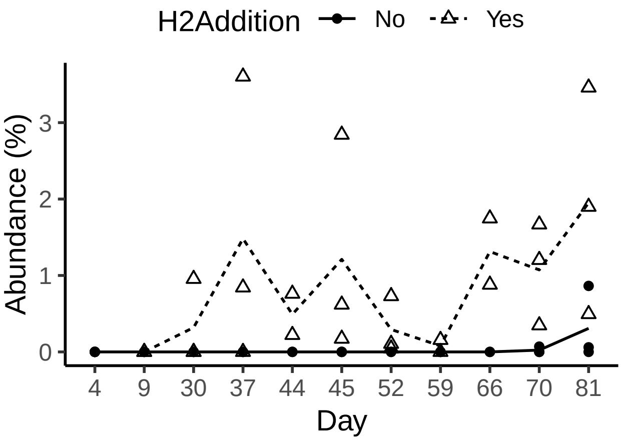

ASV62

Bacteria Firmicutes Bacilli Lactobacillales
Carnobacteriaceae Trichococcus NA

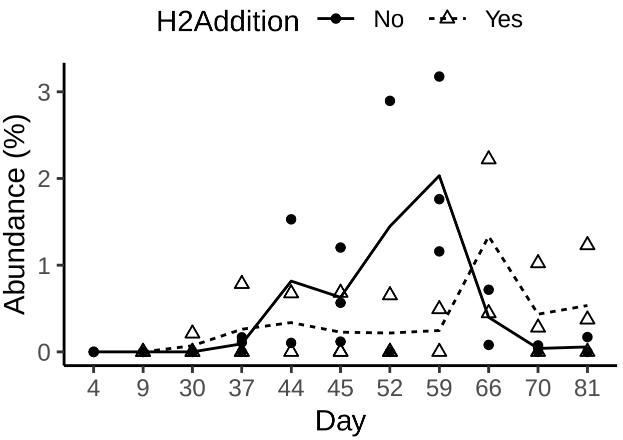

ASV65

Bacteria Synergistetes Synergistia Synergistales
Synergistaceae Syner−01 NA

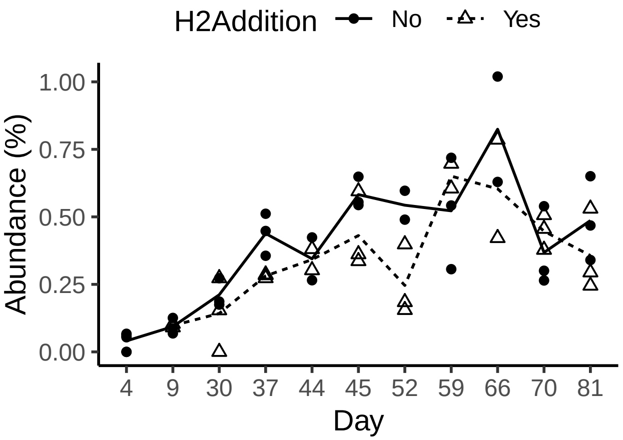

ASV63

Bacteria Thermotogae Thermotogae Petrotogales
Petrotogaceae Defluviitoga tunisiensis

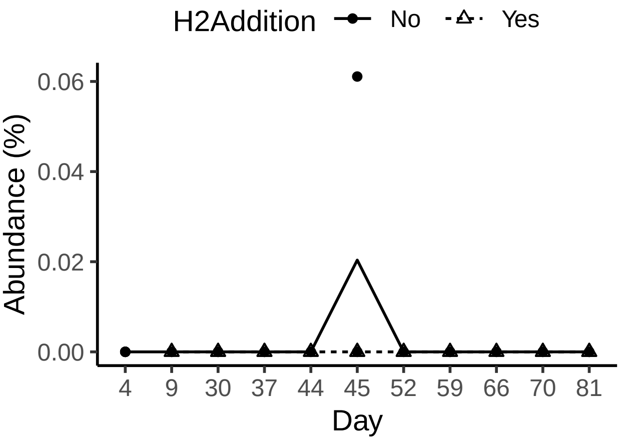

ASV66

Bacteria Proteobacteria Deltaproteobacteria Desulfovibrionales
Desulfomicrobiaceae Desulfomicrobium NA

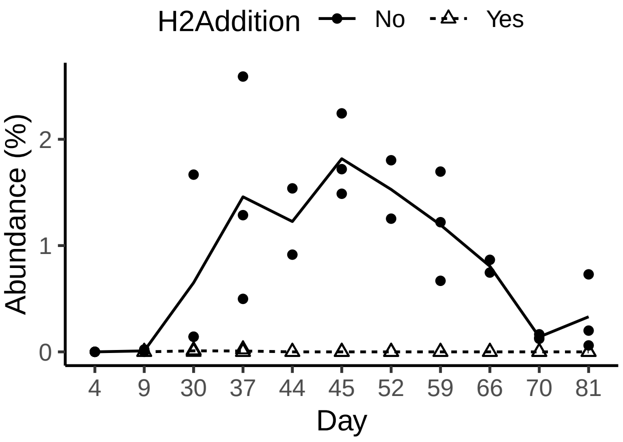

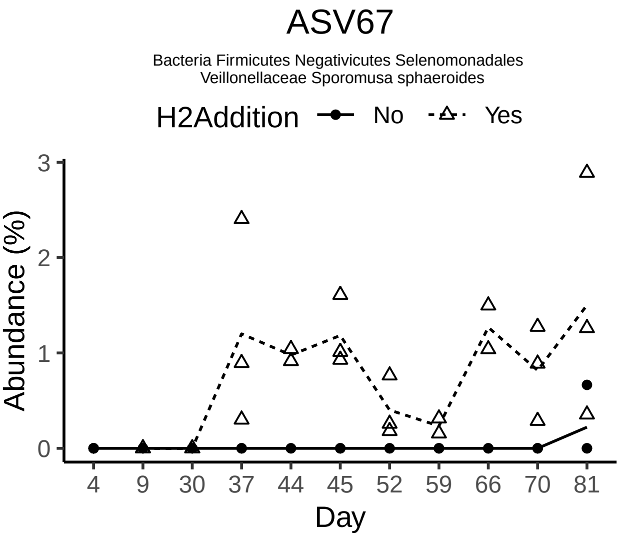
ASV67
Bacteria Firmicutes Negativicutes Selenomonadales
Veillonellaceae Sporomusa sphaeroides
H2Addition
No
Yes
Abundance (%)
0
1
2
3
4 9 30 37 44 45 52 59 66 70 81
Day

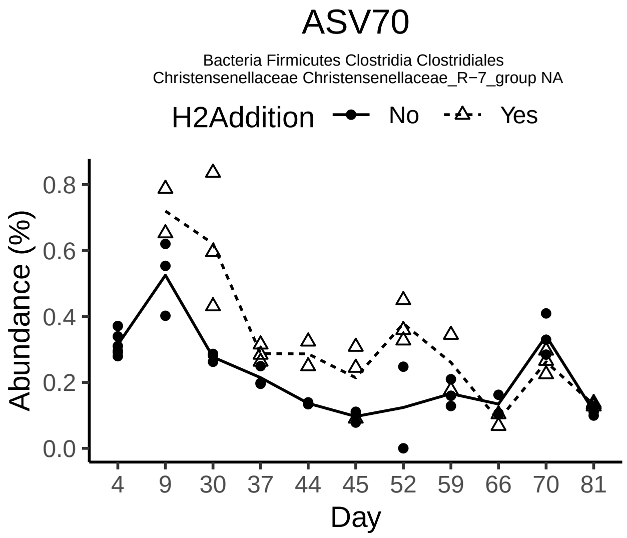
ASV70
Bacteria Firmicutes Clostridia Clostridiales
Christensenellaceae Christensenellaceae_R−7_group NA
H2Addition
No
Yes
Abundance (%)
0.0
0.2
0.4
0.6
0.8
4 9 30 37 44 45 52 59 66 70 81
Day

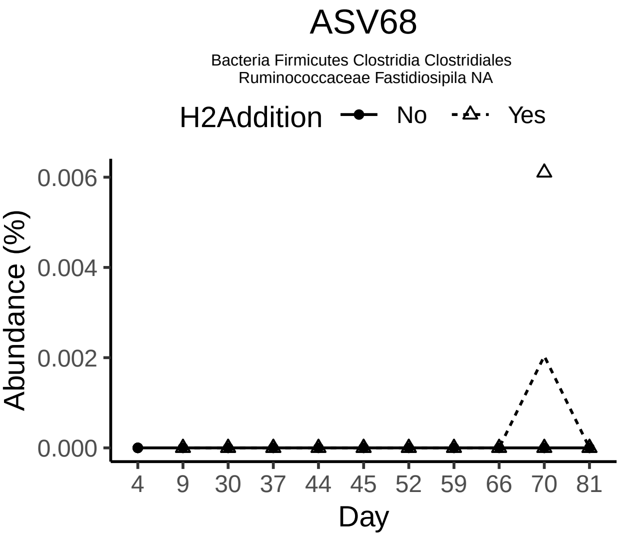
ASV68
Bacteria Firmicutes Clostridia Clostridiales
Ruminococcaceae Fastidiosipila NA
H2Addition
No
Yes
Abundance (%)
0.000
0.002
0.004
0.006
4 9 30 37 44 45 52 59 66 70 81
Day

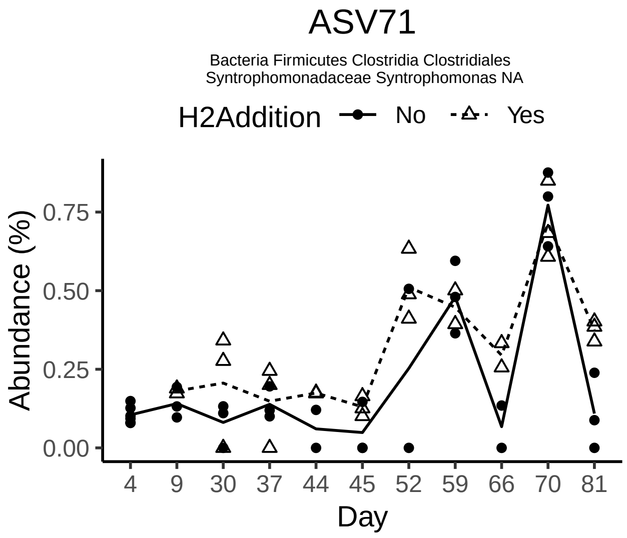
ASV71
Bacteria Firmicutes Clostridia Clostridiales
Syntrophomonadaceae Syntrophomonas NA
H2Addition
No
Yes
Abundance (%)
0.00
0.25
0.50
0.75
4 9 30 37 44 45 52 59 66 70 81
Day

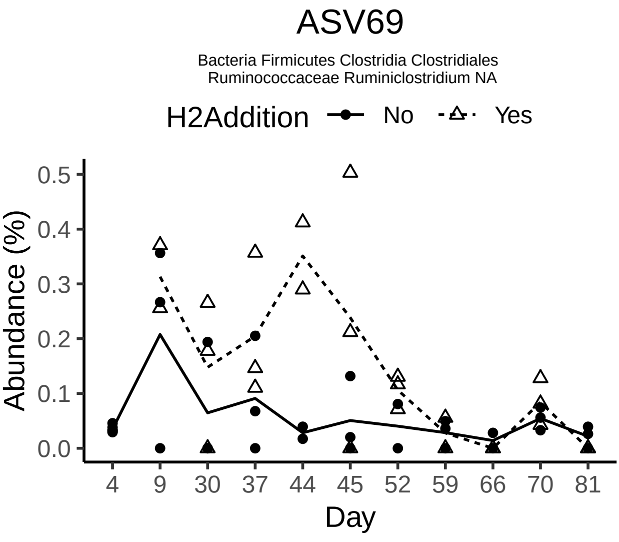
ASV69
Bacteria Firmicutes Clostridia Clostridiales
Ruminococcaceae Ruminiclostridium NA
H2Addition
No
Yes
Abundance (%)
0.0
0.1
0.2
0.3
0.4
0.5
4 9 30 37 44 45 52 59 66 70 81
Day

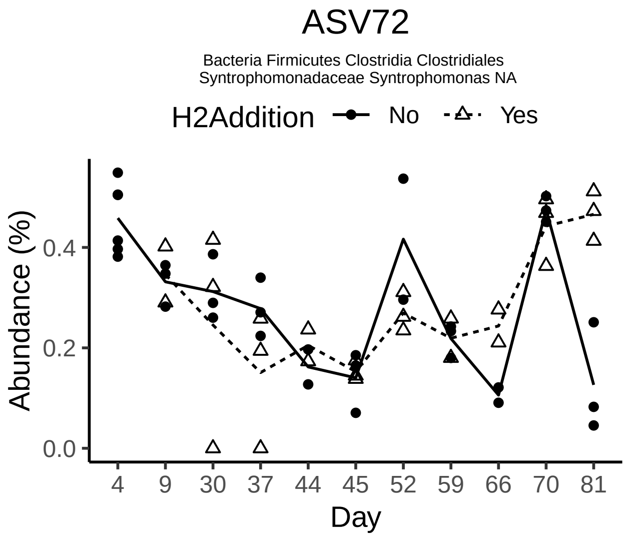
ASV72
Bacteria Firmicutes Clostridia Clostridiales
Syntrophomonadaceae Syntrophomonas NA
H2Addition
No
Yes
Abundance (%)
0.0
0.2
0.4
4 9 30 37 44 45 52 59 66 70 81
Day

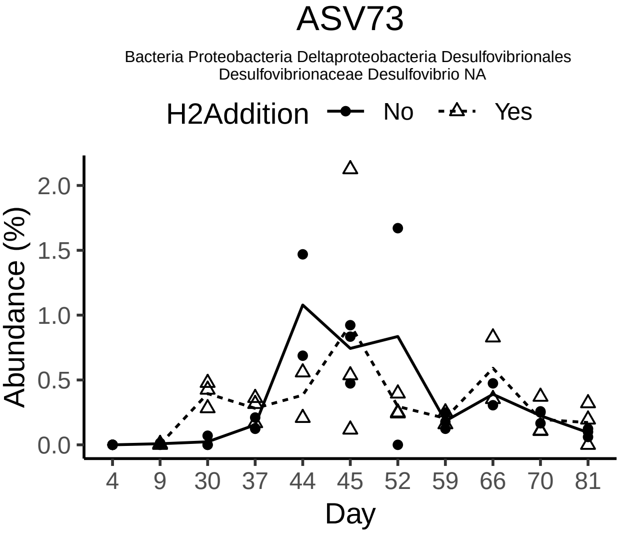
ASV73
Bacteria Proteobacteria Deltaproteobacteria Desulfovibrionales
Desulfovibrionaceae Desulfovibrio NA
H2Addition No Yes
Abundance (%)
0.0
0.5
1.0
1.5
2.0
Day
4 9 30 37 44 45 52 59 66 70 81

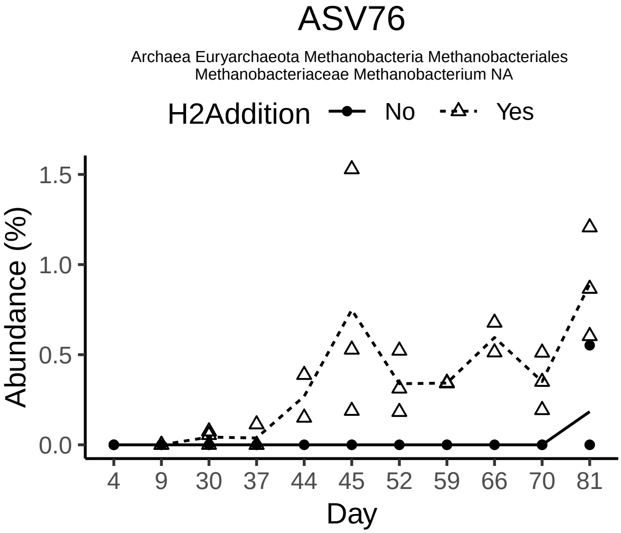
ASV76
Archaea Euryarchaeota Methanobacteria Methanobacteriales
Methanobacteriaceae Methanobacterium NA
H2Addition No Yes
Abundance (%)
0.0
0.5
1.0
1.5
Day
4 9 30 37 44 45 52 59 66 70 81

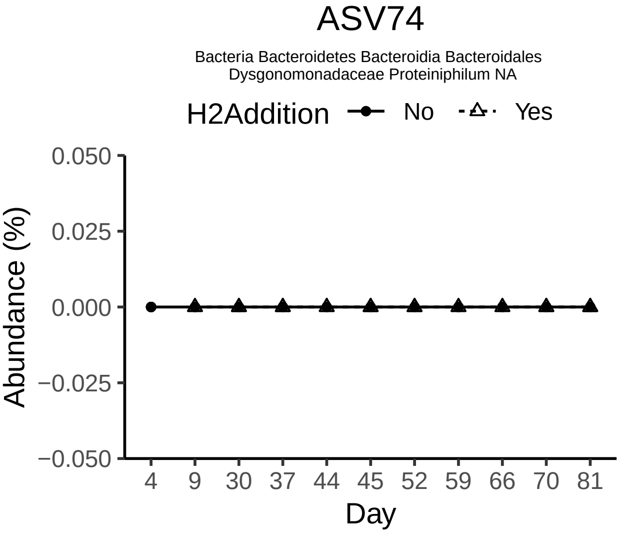
ASV74
Bacteria Bacteroidetes Bacteroidia Bacteroidales
Dysgonomonadaceae Proteiniphilum NA
H2Addition No Yes
Abundance (%)
−0.050
−0.025
0.000
0.025
0.050
Day
4 9 30 37 44 45 52 59 66 70 81

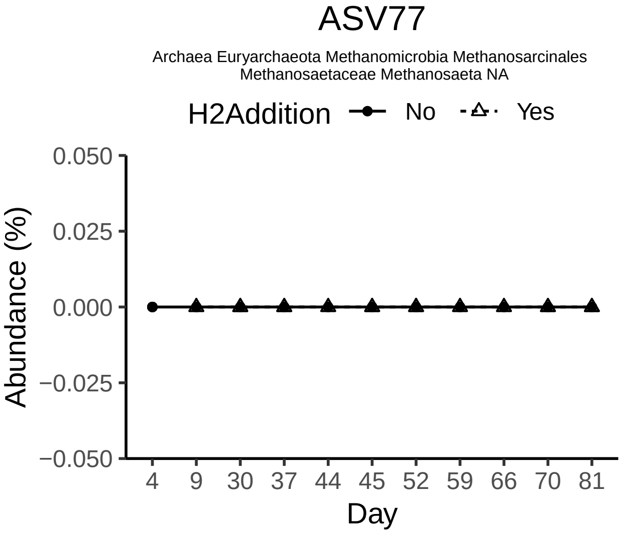
ASV77
Archaea Euryarchaeota Methanomicrobia Methanosarcinales
Methanosaetaceae Methanosaeta NA
H2Addition No Yes
Abundance (%)
−0.050
−0.025
0.000
0.025
0.050
Day
4 9 30 37 44 45 52 59 66 70 81

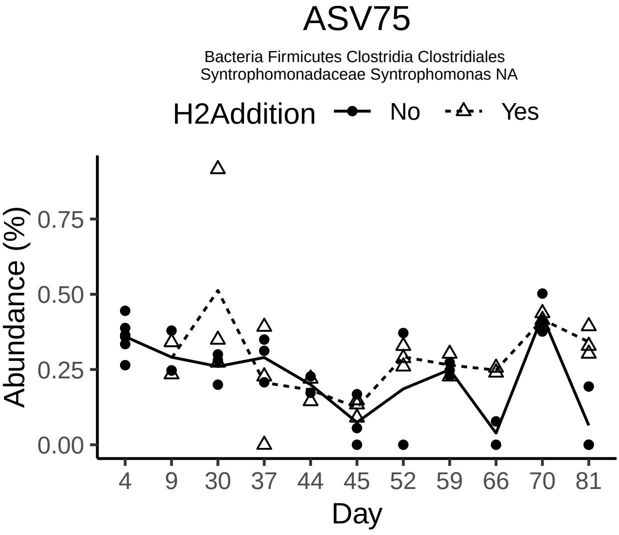
ASV75
Bacteria Firmicutes Clostridia Clostridiales
Syntrophomonadaceae Syntrophomonas NA
H2Addition No Yes
Abundance (%)
0.00
0.25
0.50
0.75
Day
4 9 30 37 44 45 52 59 66 70 81

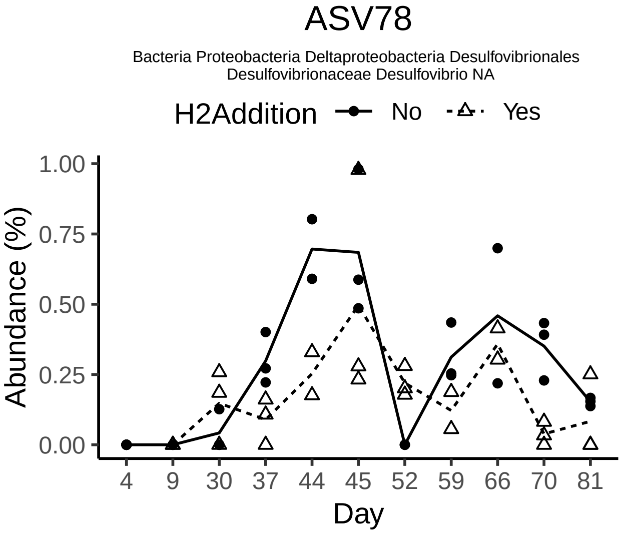
ASV78
Bacteria Proteobacteria Deltaproteobacteria Desulfovibrionales
Desulfovibrionaceae Desulfovibrio NA
H2Addition No Yes
Abundance (%)
0.00
0.25
0.50
0.75
1.00
Day
4 9 30 37 44 45 52 59 66 70 81

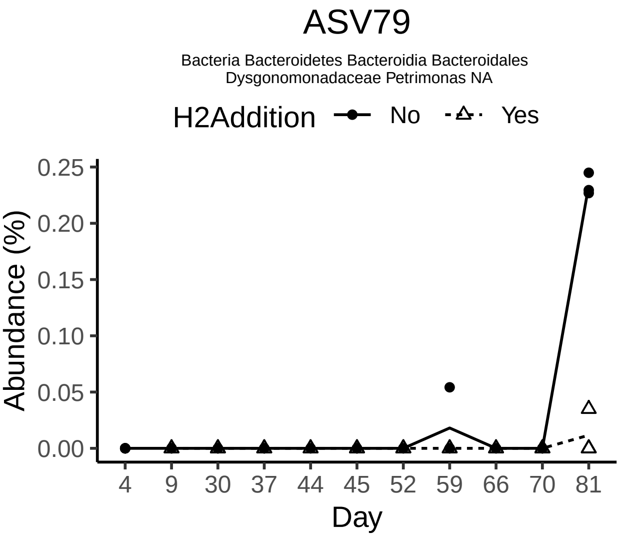
ASV79
Bacteria Bacteroidetes Bacteroidia Bacteroidales
Dysgonomonadaceae Petrimonas NA
H2Addition No Yes
Abundance (%)
0.00 0.05 0.10 0.15 0.20 0.25
Day
4 9 30 37 44 45 52 59 66 70 81

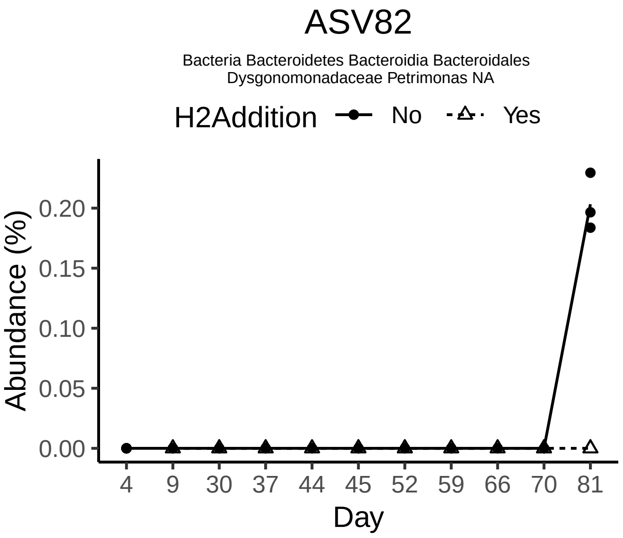
ASV82
Bacteria Bacteroidetes Bacteroidia Bacteroidales
Dysgonomonadaceae Petrimonas NA
H2Addition No Yes
Abundance (%)
0.00 0.05 0.10 0.15 0.20
Day
4 9 30 37 44 45 52 59 66 70 81

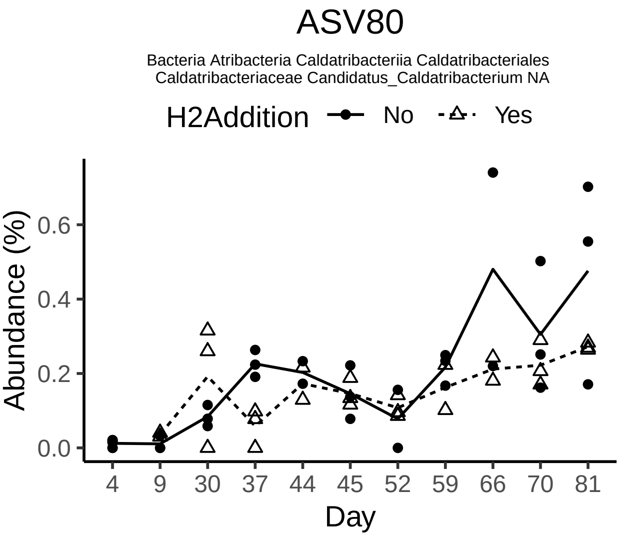
ASV80
Bacteria Atribacteria Caldatribacteriia Caldatribacteriales
Caldatribacteriaceae Candidatus_Caldatribacterium NA
H2Addition No Yes
Abundance (%)
0.0 0.2 0.4 0.6
Day
4 9 30 37 44 45 52 59 66 70 81

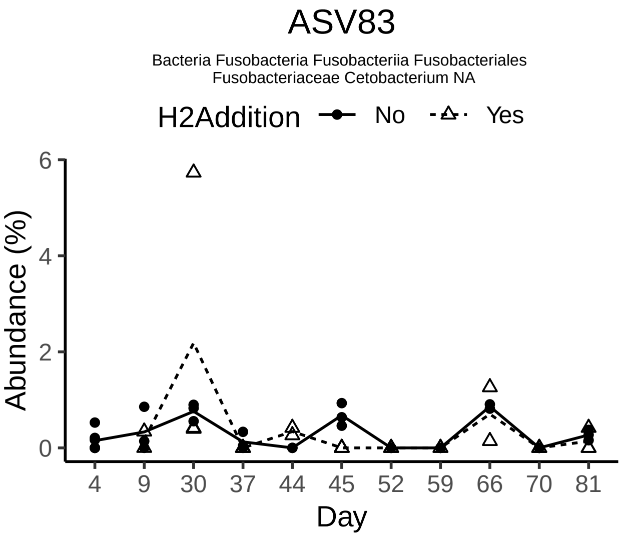
ASV83
Bacteria Fusobacteria Fusobacteriia Fusobacteriales
Fusobacteriaceae Cetobacterium NA
H2Addition No Yes
Abundance (%)
0 2 4 6
Day
4 9 30 37 44 45 52 59 66 70 81

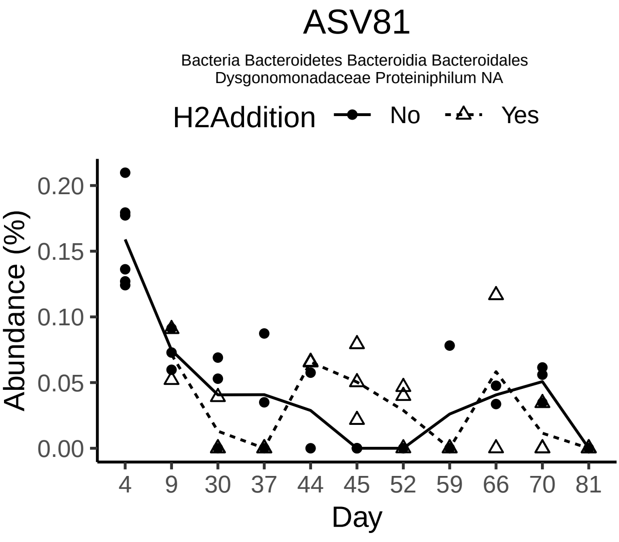
ASV81
Bacteria Bacteroidetes Bacteroidia Bacteroidales
Dysgonomonadaceae Proteiniphilum NA
H2Addition No Yes
Abundance (%)
0.00 0.05 0.10 0.15 0.20
Day
4 9 30 37 44 45 52 59 66 70 81

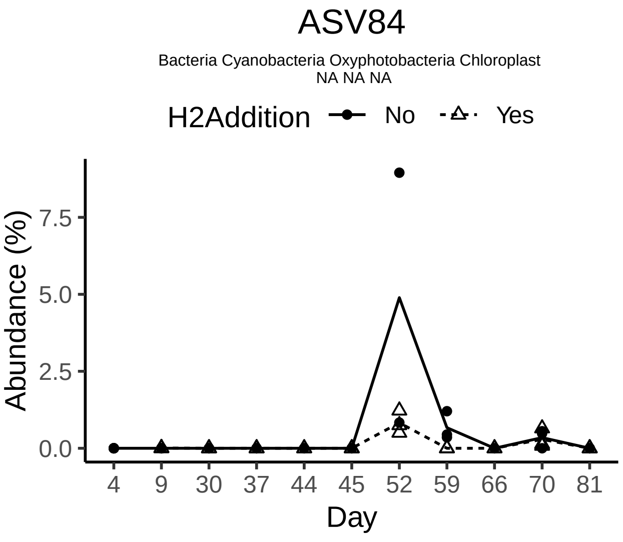
ASV84
Bacteria Cyanobacteria Oxyphotobacteria Chloroplast
NA NA NA
H2Addition No Yes
Abundance (%)
0.0 2.5 5.0 7.5
Day
4 9 30 37 44 45 52 59 66 70 81

ASV85

Bacteria Firmicutes Bacilli Lactobacillales
Carnobacteriaceae Trichococcus NA

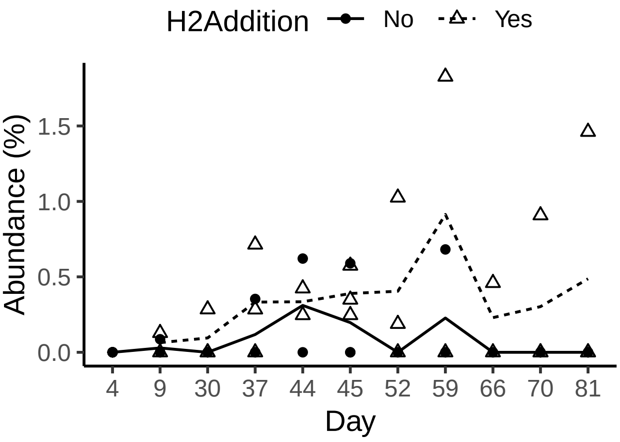

ASV88

Bacteria Firmicutes Clostridia Clostridiales
Ruminococcaceae NA NA

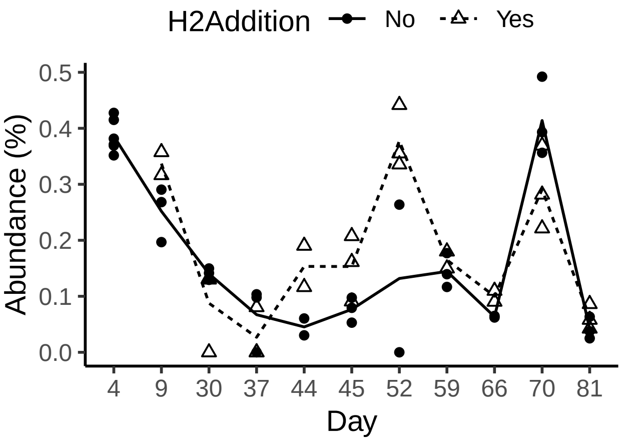

ASV86

Bacteria Bacteroidetes Bacteroidia Bacteroidales
Marinilabiliaceae Ruminofilibacter xylanolyticum

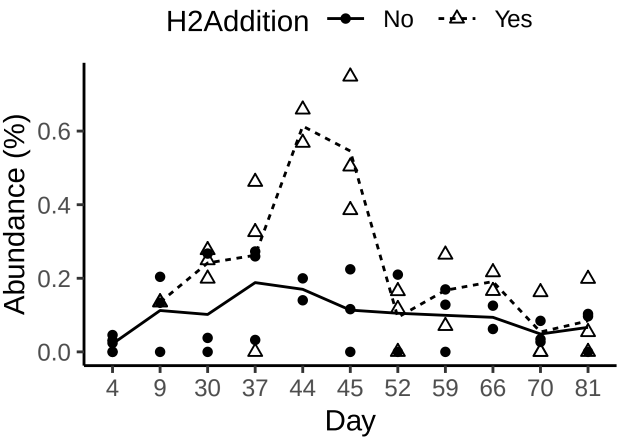

ASV89

Bacteria Bacteroidetes Bacteroidia Bacteroidales
Dysgonomonadaceae Petrimonas sulfuriphila

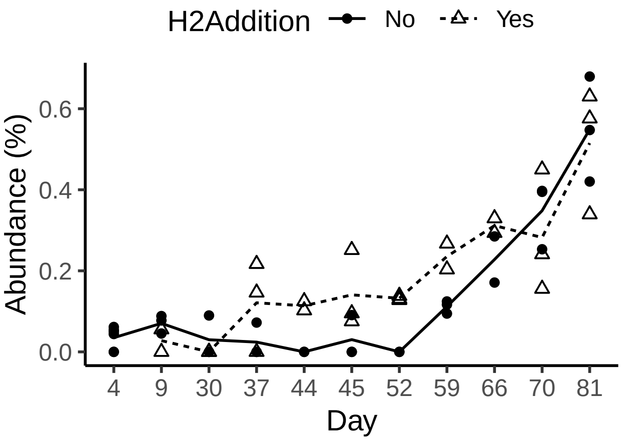

ASV87

Bacteria Firmicutes Clostridia MBA03
NA NA NA

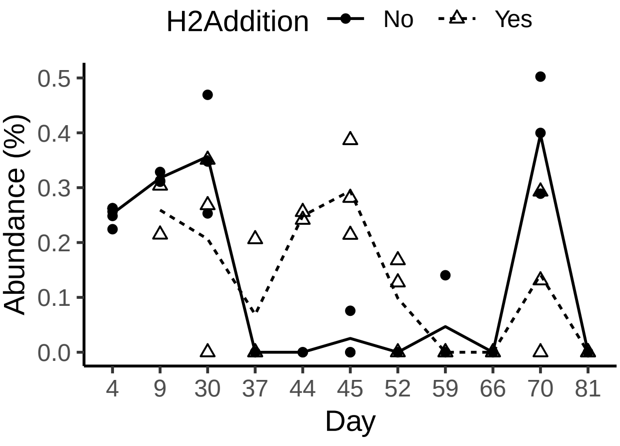

ASV90

Bacteria Proteobacteria Deltaproteobacteria Syntrophobacterales
Syntrophaceae Syntrophus aciditrophicus

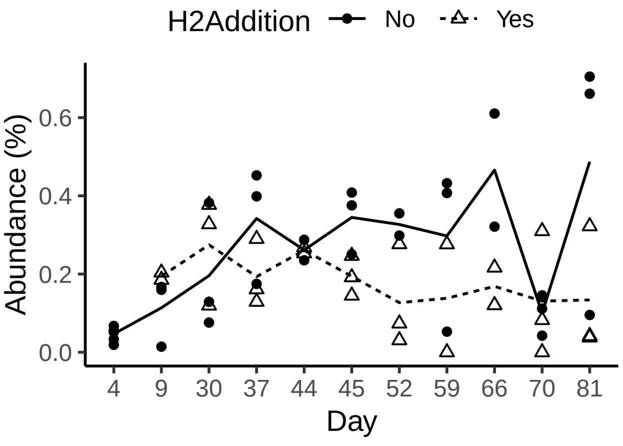

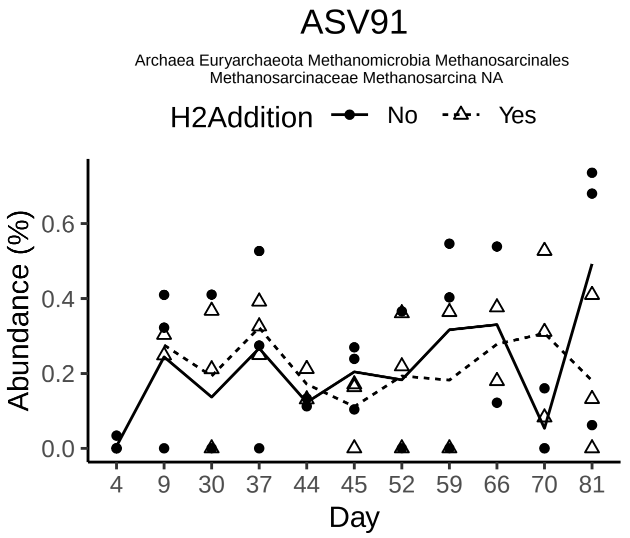
ASV91
Archaea Euryarchaeota Methanomicrobia Methanosarcinales
Methanosarcinaceae Methanosarcina NA
H2Addition
No
Yes
Abundance (%)
Day

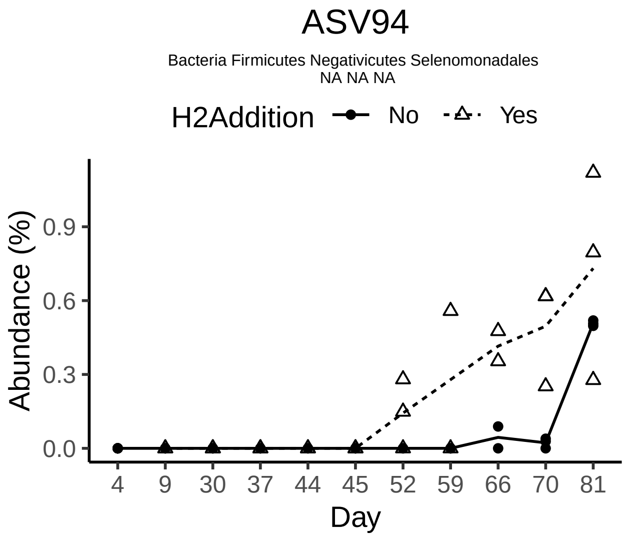
ASV94
Bacteria Firmicutes Negativicutes Selenomonadales
NA NA NA
H2Addition
No
Yes
Abundance (%)
Day

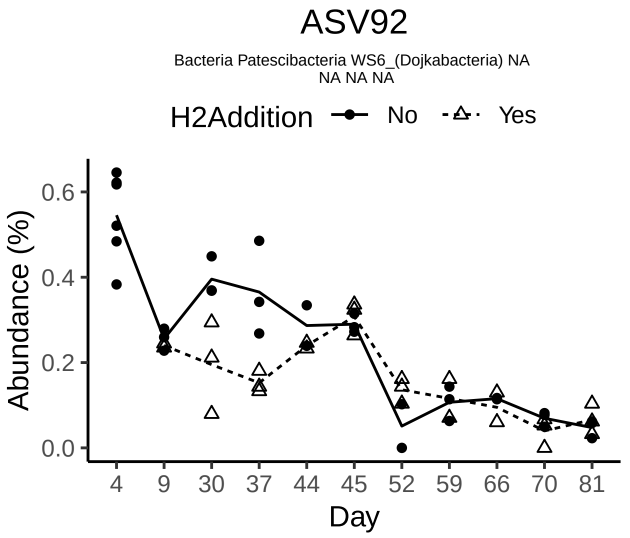
ASV92
Bacteria Patescibacteria WS6_(Dojkabacteria) NA
NA NA NA
H2Addition
No
Yes
Abundance (%)
Day

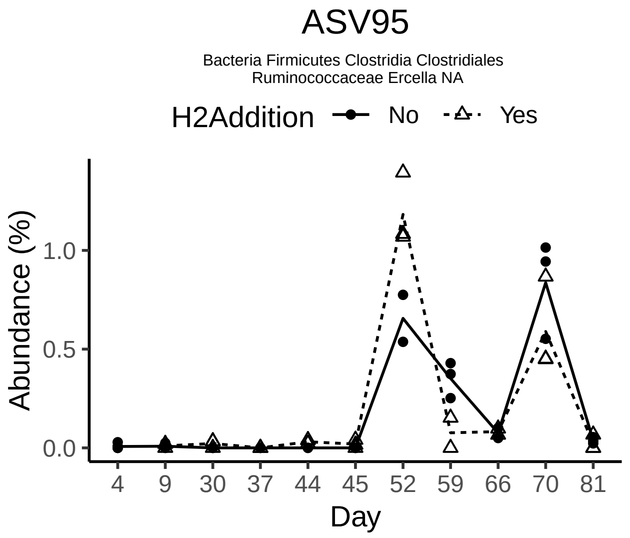
ASV95
Bacteria Firmicutes Clostridia Clostridiales
Ruminococcaceae Ercella NA
H2Addition
No
Yes
Abundance (%)
Day

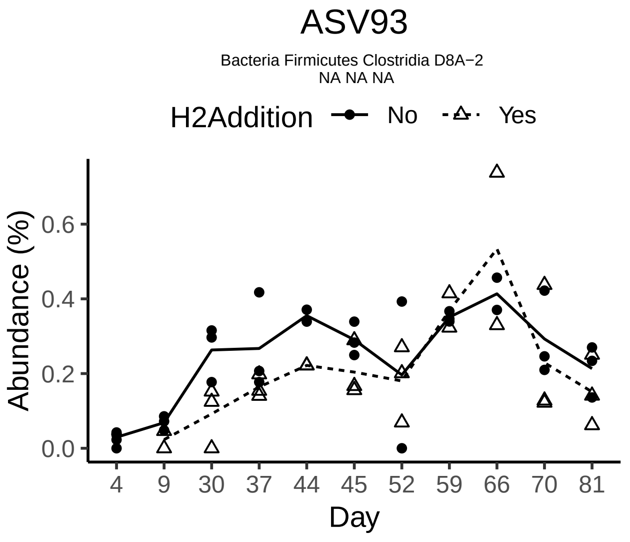
ASV93
Bacteria Firmicutes Clostridia D8A−2
NA NA NA
H2Addition
No
Yes
Abundance (%)
Day

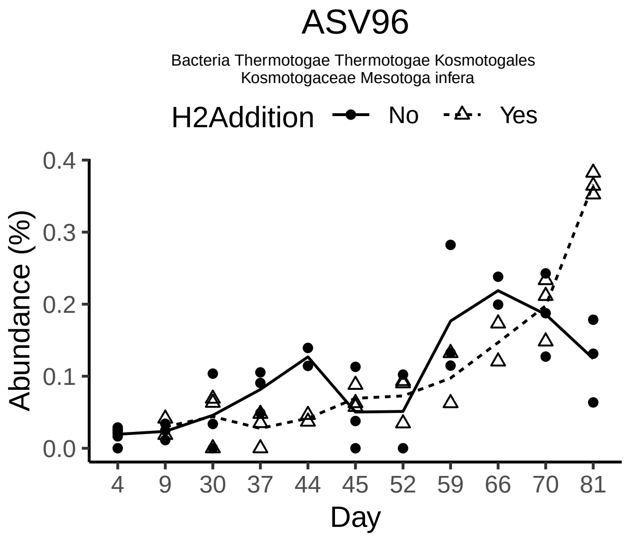
ASV96
Bacteria Thermotogae Thermotogae Kosmotogales
Kosmotogaceae Mesotoga infera
H2Addition
No
Yes
Abundance (%)
Day

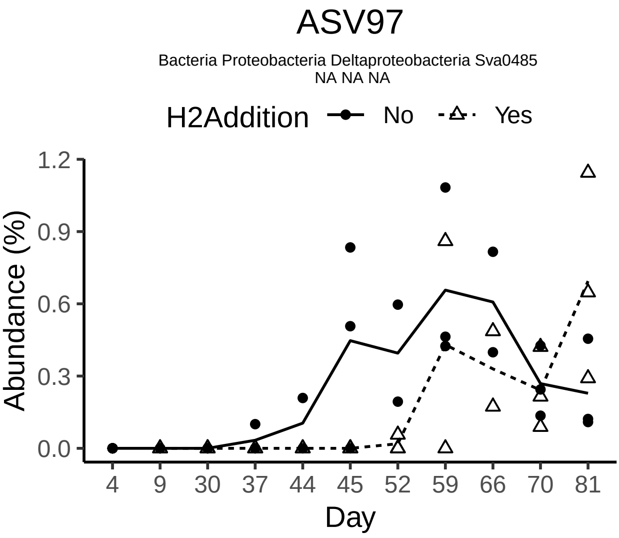
ASV97
Bacteria Proteobacteria Deltaproteobacteria Sva0485
NA NA NA
H2Addition No Yes
Abundance (%)
1.2
0.9
0.6
0.3
0.0
4 9 30 37 44 45 52 59 66 70 81
Day

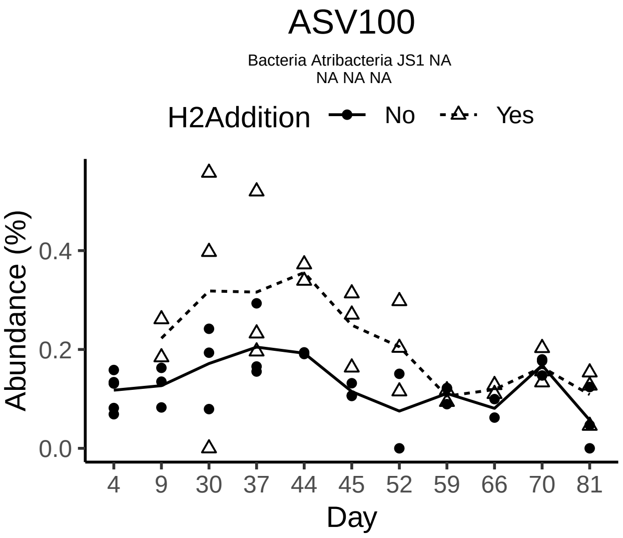
ASV100
Bacteria Atribacteria JS1 NA
NA NA NA
H2Addition No Yes
Abundance (%)
0.4
0.2
0.0
4 9 30 37 44 45 52 59 66 70 81
Day

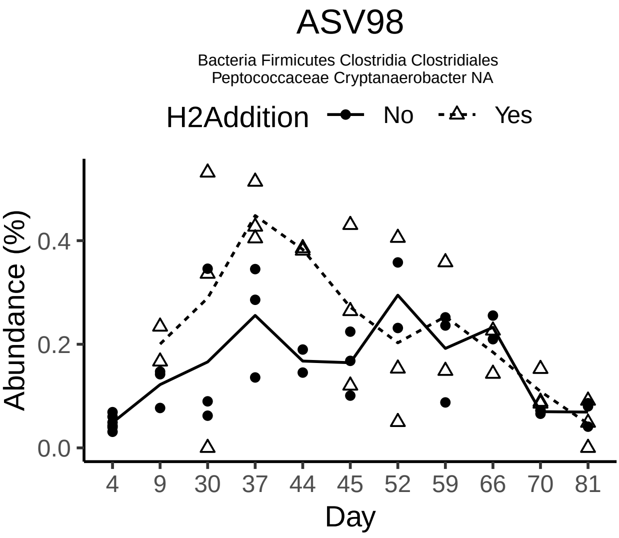
ASV98
Bacteria Firmicutes Clostridia Clostridiales
Peptococcaceae Cryptanaerobacter NA
H2Addition No Yes
Abundance (%)
0.4
0.2
0.0
4 9 30 37 44 45 52 59 66 70 81
Day

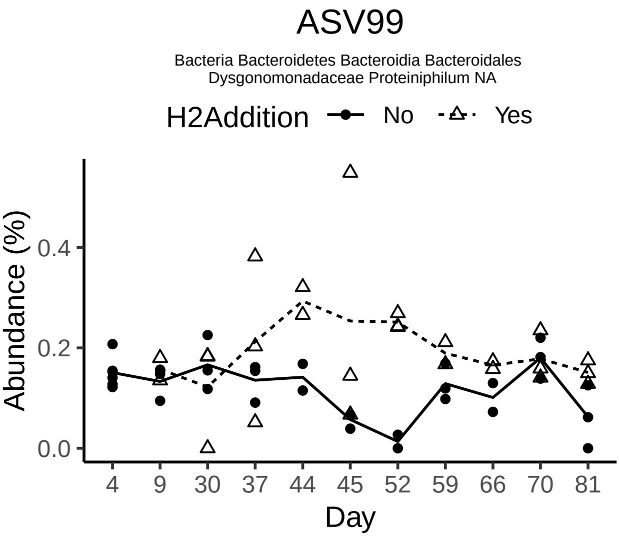
ASV99
Bacteria Bacteroidetes Bacteroidia Bacteroidales
Dysgonomonadaceae Proteiniphilum NA
H2Addition No Yes
Abundance (%)
0.4
0.2
0.0
4 9 30 37 44 45 52 59 66 70 81
Day
